# Genome sequence analysis of the beneficial *Bacillus subtilis* PTA-271 isolated from a *Vitis vinifera* (cv. Chardonnay) rhizospheric soil: assets for sustainable biocontrol

**DOI:** 10.1186/s40793-021-00372-3

**Published:** 2021-01-29

**Authors:** Catarina Leal, Florence Fontaine, Aziz Aziz, Conceiçao Egas, Christophe Clément, Patricia Trotel-Aziz

**Affiliations:** 1 SFR Condorcet – FR CNRS 3417, University of Reims Champagne-Ardenne, Induced Resistance and Plant Bioprotection (RIBP) – EA 4707, BP1039, Cedex 2, F-51687 Reims, France; 2 UC-Biotech_CNC, Biocant Park, Biotechnology Innovation Center, P-3060-197 Cantanhede, Portugal

**Keywords:** Genome draft, Beneficial bacterium, *Bacillus subtilis* PTA-271, Grapevine trunk diseases, Grey mold, Wide protective spectrum, Sustainable biocontrol

## Abstract

**Background:**

*Bacillus subtilis* strains have been widely studied for their numerous benefits in agriculture, including viticulture. Providing several assets, *B. subtilis* spp. are described as promising plant-protectors against many pathogens and as influencers to adaptations in a changing environment. This study reports the draft genome sequence of the beneficial *Bacillus subtilis* PTA-271, isolated from the rhizospheric soil of healthy *Vitis vinifera* cv. Chardonnay at Champagne Region in France, attempting to draw outlines of its full biocontrol capacity.

**Results:**

The PTA-271 genome has a size of 4,001,755 bp, with 43.78% of G + C content and 3945 protein coding genes. The draft genome of PTA-271 putatively highlights a functional swarming motility system hypothesizing a colonizing capacity and a strong interacting capacity, strong survival capacities and a set of genes encoding for bioactive substances. Predicted bioactive compounds are known to: stimulate plant growth or defenses such as hormones and elicitors, influence beneficial microbiota, and counteract pathogen aggressiveness such as effectors and many kinds of detoxifying enzymes.

**Conclusions:**

Plurality of the putatively encoded biomolecules by *Bacillus subtilis* PTA-271 genome suggests environmentally robust biocontrol potential of PTA-271, protecting plants against a broad spectrum of pathogens.

**Supplementary Information:**

The online version contains supplementary material available at 10.1186/s40793-021-00372-3.

## Background

*Bacillus subtilis* is a Gram-positive endospore-forming bacterium from *Bacillus* genera considered as a promising plant beneficial organism that can survive in the soil for extended time periods under harsh environmental conditions [1]. Benefits of species from the *Bacillus* group are well described in many sectors of industry, agriculture and viticulture [2]. Focusing on the *B. subtilis* species, it has been described to provide plants with a broad range of benefits that include induced systemic resistance (ISR) upon pathogen attacks, growth promotion, or the direct control of plant pathogens [3–6].

Primed defenses during ISR are regulated either by jasmonic acid (JA) and ethylene (ET) signaling or by salicylic acid (SA) signaling [7–10]. Beneficial microorganisms may modulate the plant hormonal balance by either altering hormone synthesis or by producing similar hormones or their precursors (ET, SA, auxins, gibberellins, cytokinins, polyamines…) [8]. Numerous bacterial elicitors of ISR are also reported in several plant species, such as exopolysaccharides (EPS), lipopolysaccharides (LPS), siderophores such as the iron-regulated pyoverdin, iron, flagella, biosurfactants, N-acyl-L-homoserine lactone, N-alkylated benzylamine and volatile compounds [8, 9, 11, 12]. Some of these have already been identified in species of *B. subtilis* or *Bacillus* genera [8, 11, 13, 14]. Changes in the phytohormonal-balance also impact plant growth and development, since the reduction of ET may promote plant growth [8, 15, 16]. Microbiota support plant growth and development by modulating nutrient availability through mineralization and chelation, as well as through the production of volatile compounds that support biocontrol [17, 18]. Efficient beneficial effects of *Bacillus spp*. also assume direct and indirect bacterium and microbiota preservation, upon abiotic and biotic stressful conditions [8, 19]. When biocontrol agents protect themselves through extrusion transporters, detoxifying enzymes, quenching enzymes and pathogen homologous enzymes, they also contribute indirectly to plant protection [8]. Finally, *B. subtilis* produces an extensive range of antimicrobial molecules, chelators and lytic enzymes that limit pathogen fitness and aggressiveness [20]. According to literature, these beneficial molecules include ribosomally synthesized antimicrobial peptides (RP, including the post-translationally modified peptides RiPP), non-ribosomally synthesized peptides (NRP), polyketides (PK), as well as other uncommon antimicrobial volatile compounds (the inorganic and organic ViCs and VOCs, respectively) and terpenoid secondary metabolites as listed in Table 1. Individual strain specificities may thus impact both biochemical conditions and species ratios, and in turn interactions among complex microbial communities and their hosts.

**Table 1:**
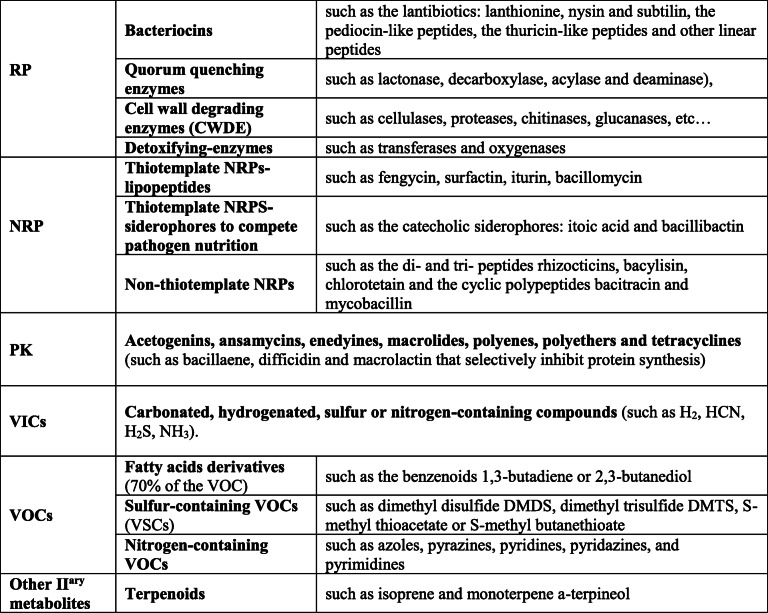
*Bacillus subtilis* known antimicrobial molecules, chelators and lytic enzymes [6, 20, 21]

*RP*   Ribosomally synthesized antimicrobial peptides (including the post-translationally modified peptides RiPP), *NRP*   Non-ribosomally synthesized peptides, *PK*   Polyketides, *VIC*   Inorganic antimicrobials volatile compounds, *VOC*   Organic antimicrobials volatile compounds

Focusing on *B. subtilis* PTA-271, its protective effect has been published in grapevine against *Neofusicoccum parvum* and *Botrytis cinerea* [3–5], the causal agents of Botryosphaeria dieback and grey mold respectively. The ability of *B. subtilis* species to sporulate in order to resist climate changes and common disinfectants [1], combined with the fact that *B. subtilis* PTA-271 is a non-pathogenic species, make this microorganism suitable to control a wide spectrum of pathogens among which the most economically significant grapevine trunk disease (GTD) pathogens currently lack of efficient control strategies [3, 22]. In this study, we report the draft genome sequence of the *B. subtilis* strain PTA-271, analyze and compare with other known *Bacillus* strains sequences, to expand our knowledge of *B. subtilis* PTA-271 benefits, as well as design efficient and sustainable biocontrol strategies for viticulture.

## Methods

### *B. subtilis* PTA-271 GENERAL INFORMATION AND FEATURES

*B. subtilis* PTA-271 was isolated in 2001 (Table 2) from the rhizospheric soil of healthy Chardonnay grapevines (*V. vinifera* L., cv Chardonnay) from a vineyard located in Champagne (Marne, France). Rhizospheric samples were directly suspended in a sterile 0.85% NaCl solution (1 g of soil: 10 ml of NaCl) and bacterial isolates were obtained by serial dilutions of the soil samples (10^7^, 10^3^, 10^2^ cfu/g soil) in triplicate onto LB-agar (Luria–Bertani-agar), King’s B-agar and glycerol–arginine-agar plates by incubating at 30 °C for 24–72 h. All different colonies were then re-isolated on LB-agar, cultured in LB at 30 °C for 24 h and screened for their protective role against *Botrytis cinerea* by using grapevine plantlet leaf assays pretreated with bacterium [4]. Selected biocontrol microorganisms were then identified, calculated to establish the density formula and stored in a sterile 25% glycerol solution at − 80 °C for complementary purposes. The classification and general features of *B. subtilis* PTA-271 are in Table 2. The taxonomic information for this strain was already described by Trotel-Aziz et al. (2008) [4] and remains unaltered to this date.

**Table 2:**
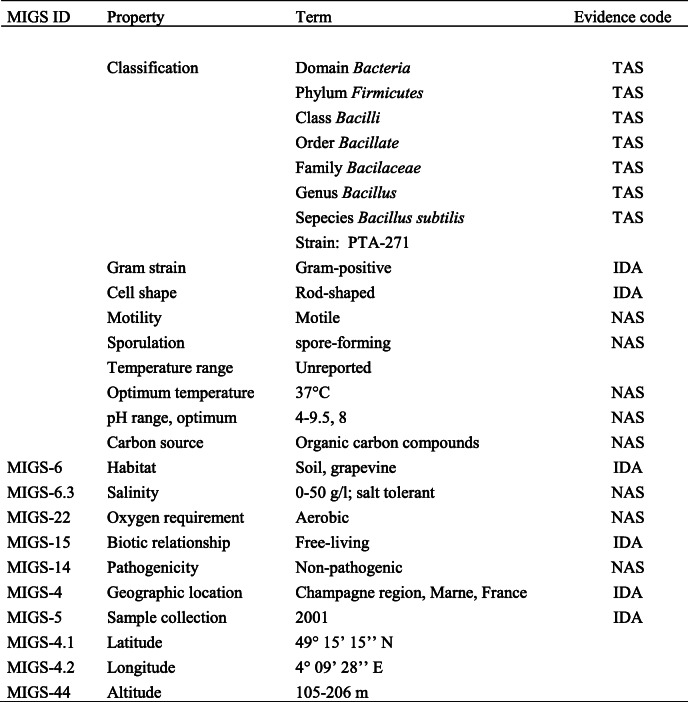
Classification and features of *Bacillus subtilis* PTA-271 according to MIGS recommendations [23]

^a^Evidence codes (from the Gene ontology project [58] – *IDA* Inferred from Direct assay, *TAS* Traceable Author Statement (i.e., a direct report exists in the literature), *NAS* Non-traceable Author Statement (i.e., not directly observed from the living, isolated sample, but based on a generally accepted property for the species, or anecdotal evidence)

### *B. subtilis* PTA-271 GENOMIC SEQUENCING INFORMATION

#### Genome project history

*B. subtilis* PTA-271 was selected for sequencing due to its efficient capacity to protect grapevine against several pathogens with distinct lifestyles such as *Botrytis cinerea* and *Neofusicoccum parvum* [3–5]. This beneficial microorganism can not only modulate grapevine defenses, but also antagonize the growth of pathogens and detoxify aggressive molecules. These beneficial bacteria provide protection against a broad spectrum of pathogens, due to its genetic traits of physical and chemical tolerance (endospore forming, withstand large pH and salinity range, Table 2). Altogether, there are advantages to sequence the *B. subtilis* PTA-271 genome to better understand its key beneficial levers and develop better sustainable biocontrol strategies regardless of field conditions or soil parameters (pH, salinity, etc.).

The whole genome shotgun project has been deposited at DDBJ/ENA/GenBank under the accession JACERQ000000000. The version described in this paper is version JACERQ000000000 and all related information is represented in Table 3.

**Table 3:**
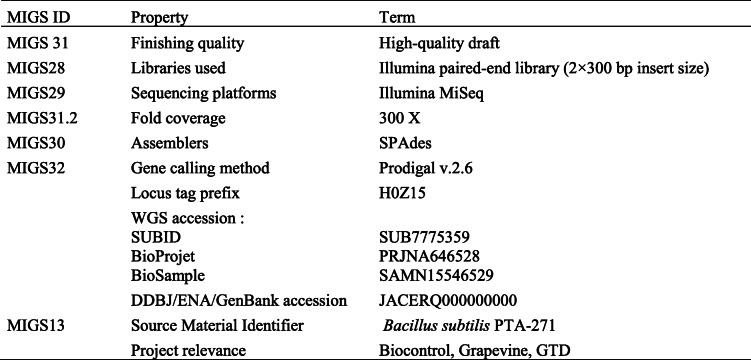
*Bacillus subtilis* PTA-271 genomic sequencing information

#### Genomic DNA preparation

Genomic DNA of *B. subtilis* PTA-271 was extracted using the Wizard® Genomic DNA Purification kit (Promega), from the pellet of a 1 mL-overnight culture incubated at 28 °C in LB medium. DNA integrity was confirmed on a 0.65% agarose gel electrophoresis in TAE buffer. DNA concentration and quality were read from 1 μL of DNA with the NanoDrop-ONE spectrophotometer (Ozyme).

#### Library preparation and genome sequencing

DNA library for bacterial genome sequencing was prepared from 0.5 nanograms of high-quality genomic DNA using the Nextera XT DNA Sample Preparation Kit (Illumina, San Diego, USA) and sequenced using paired-end (PE) 2 × 300 bp on the MiSeq® Illumina® platform at Genoinseq (Cantanhede, Portugal). All the procedures were performed according to standard manufacturer protocols.

#### Genome assembly and annotation

Sequenced reads were demultiplexed automatically by the Illumina® Miseq® sequencer using the CASAVA package (Illumina, San Diego, USA) and quality-filtered with Trimmomatic version 0.30 [24]. High-quality adapter-free reads were assembled with SPAdes version 3.9.0 [25] and contigs with size < 500 bp or coverage lower 10x were removed from the assembly. Assembly metrics were calculated with Quast version 4.6.1 [26]. Contigs were checked for contamination and completeness using CheckM 1.0.9 [27]. Coding gene predictions were made with Prodigal version 2.6 [28], rRNA and tRNA genes were detected using Barrnap version 0.8 and CRISPR regions were detected by Minced version 0.2.0. Coding gene annotation was performed with Prokka version 1.12 [29] using the following repositories: SwissProt (The UniProt Consortium, 2017), HAMAP [30], TIGRFAMs [31] and Pfam [32]. Coding genes were also annotated for Pathway using KEGG [33], for peptidases using MEROPS [34] and for carbohydrate-active enzymes with dbCaN [35].

## Results and discussion

### *B. subtilis* PTA-271 GENOME PROPERTIES AND COMPARISON WITH OTHER BACILLUS STRAINS

**Fig. 1:**
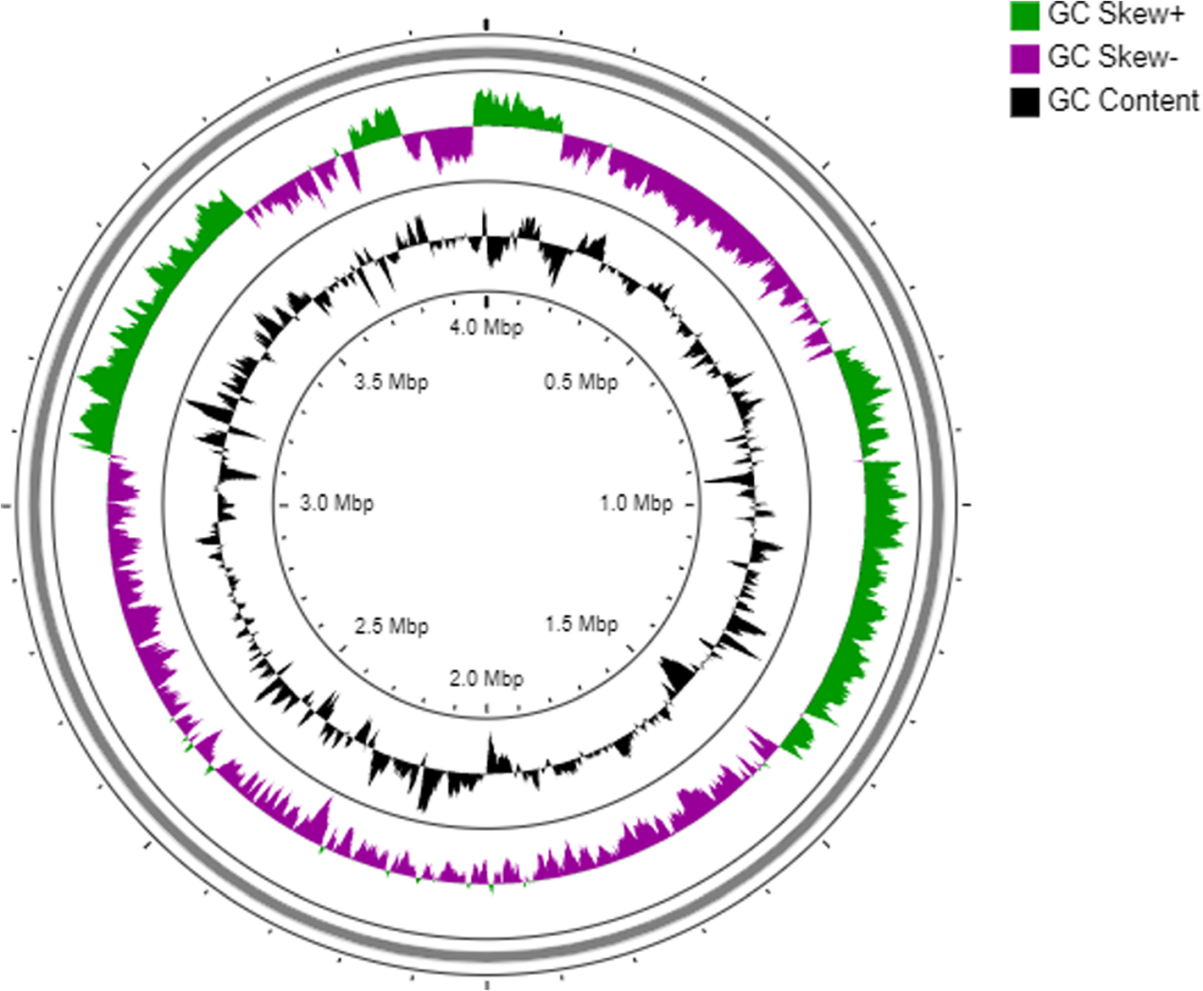
Circular map of the *Bacillus subtilis* PTA-271 genome. Map generated with CGView server [36]

The general features of *B. subtilis* PTA-271 are in Table 4 and Fig. 1, performed using Artemis version 16.0.0. The draft genome sequence of *B. subtilis* PTA-271 presented an estimated genome size of 4,001,755 bp divided in 20 contigs. The G + C content of this sequence was 1,751,999 bp, representing about 43.78% of the whole genome. Genome analysis showed that *B. subtilis* PTA-271 contained 4038 genes, among which 3945 (97.69%) were protein coding genes. This genome draft predicts 92 RNA genes among which 11 rRNA genes were identified and no CRISPR repeats. From 4,001,755 bp of the genome size, 3,550,299 bp correspond to coding genes representing 88.73% of the whole genome. From this, 3440 genes had function prediction, 3183 were assigned to the COG categories described in Table 5, and 3517 genes had Pfam domain descriptions.

**Table 4:**
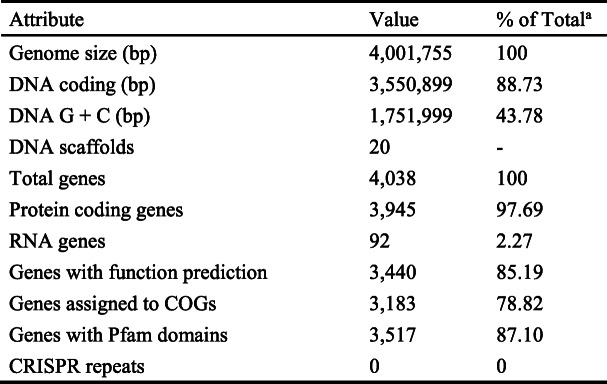
Genome statistics

^a^ The total is based on either the size of genome in base pairs or the total number of genes in the predicted genome

**Table 5:**
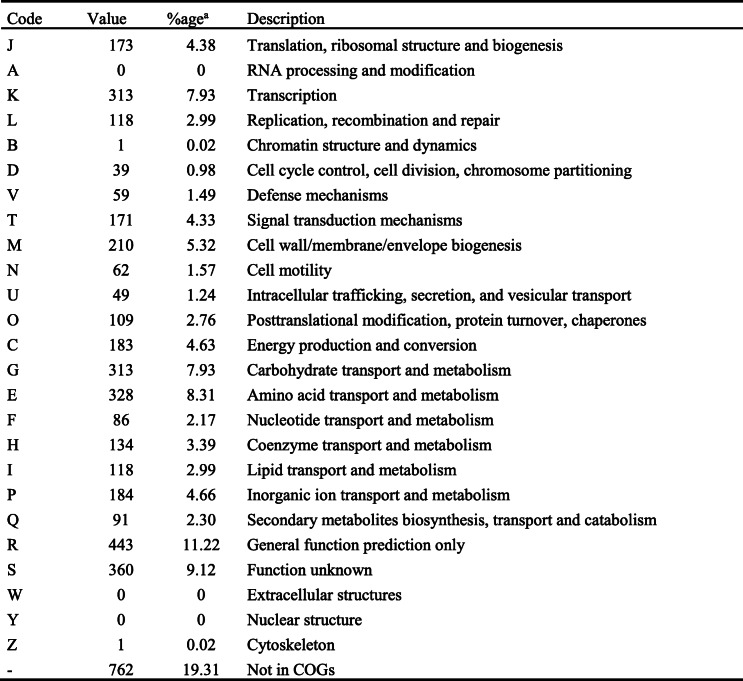
Number of genes associated with general COG functional categories

^a^ The total is based on the total number of protein-coding genes in the genome

### *B. subtilis* PTA-271 ASSETS FOR PLANT SUSTAINABLE BIOCONTROL

*Bacillus* species offer a broad range of benefits to plants: (1) plant growth promotion, (2) induced systemic plant defenses and protection against pathogens, and (3) prevention of pathogen fitness or aggressiveness, by producing many compounds able to interact with the host plants, the pathogens or their tripartite intricate communication. Considering this, the genome analysis of *B. subtilis* PTA-271 tried to highlight some useful characteristics directly or indirectly beneficial for a sustainable plant protection against a broad spectrum of pathogens.

#### Motility and adhesion: assets for plant root colonization

Motility of a bacterium is due to the flagellum, enabling it to move towards a vital nutrient source (chemotaxis). In this sense, *B. subtilis* PTA-271 contains genes (Supplementary Table S1) putatively encoding for flagella maintenance (*flh* genes) and chemotaxis (*che* genes). Once reaching a comfortable area, adhesion is due to bacterium pili, allowing the initiation of biofilm formation where both chemotaxis and gene exchanges among microorganisms of microbiota can be amplified.

*B. subtilis* spp. are also described for their strong swarming motility [37]. The gene *swrC* putatively encoding for swarming motility protein is predicted in the genome of *B. subtilis* PTA-271 (Supplementary Table S1). Swarming motility requires the production of functional flagella, pili and surfactant to reduce surface tension.

Motilities and adhesion are considered advantageous characters for a successful host colonization and *B. subtilis* spp. are already described to grow in biofilm mode involved in root colonization [38]. To this end, the transcription factors (TF) Spo0A and AbrB were described as positive and negative regulators of biofilm formation, respectively [39]. Genes putatively encoding for these 2 TFs are also predicted in the genome of *B. subtilis* PTA-271 as *Spo0A* and *AbrB* (Supplementary Tables S1 and S2).

Beneficial microorganisms that successfully colonize the plant, particularly by the root system, would be advantageous, both for plant growth promotion and for plant biocontrol [40, 41].

#### Biofertilizing and morphogenic effects: assets for plant vigor

Plant nutrition depends on soil retention capacity of minerals and nutrient availabilities, thus both on chelating process, mineralization by decomposers and minerals bioavailability towards the plant consumer. Upon nitrogen starvation, some bacteria are described to upregulate the *ure* gene cluster, since urea is an easy nitrogen source. Such *ure* genes are predicted in *B. subtilis* PTA-271 genome (*ureA*, *ureB*, *ureC*). This cluster of genes is known to be controlled by the global nitrogen-regulatory protein *TnrA*, also predicted in *B. subtilis* PTA-271 genome (Supplementary Table S2). Regarding other nutrient access due to phosphate-solubilizing bacteria (PSB) [6, 42, 43], genes encoding for proteins involved in the production of gluconic acid and precursor of citric acid are also predicted in the genome of *B. subtilis* PTA-271 (S19-40_03830, S19-40_03828). Organic acids may lower the soil pH to solubilize phosphate and thus increase its availability to the plant [42]. Bacterial secondary metabolites (PyrroloQuinoline Quinone, PQQ) are also known to control gluconic acid production [44], and *B. subtilis* PTA-271 has 3 genes predicted to be related to PQQ production *pqqL, pqqF and pqqC* [45]. Additionally, *B. subtilis* PTA-271 contains the phytase gene *phy*, described in the other *Bacillus spp*. to encode for phosphatases able to hydrolyze organic complex in order to liberate phosphate and make it available for plants [46]. Iron is another very important nutrient for plant growth and development. *B. subtilis* PTA-271 possesses the *fur* gene (Supplementary Table S2) described in the literature to encode for a regulatory protein coordinating the homeostasis of iron uptake depending on its availability in the soil [47]. Regarding soils containing abundant ferric form (Fe^3+^) which is poorly available to plants, the literature described bacteria producing siderophores with high specificity and affinity for iron, capable of binding, extracting and transporting iron near the plant roots [48]. *B. subtilis* PTA-271 genome also predicted the production of such siderophores, namely the catecholic siderophore 2,3-dihydroxybenzoate-glycine-threonine trimeric ester bacillibactin encoded by 5 genes (*dhbA* to *dhbF*). Surfactants produced by beneficial bacteria also contribute to increase the availability of hydrophobic nutrients. In this sense, *B. subtilis* PTA-271 is suspected to produce surfactin (with *srfAA* to *srfAD*), a powerful biosurfactant due to its amphiphilic nature that strongly anchor with lipid layers, interfering with the structure of biological membranes [49].

Plant root morphology is also described to impact nutrient uptake and thus plant growth due to the stimulation of lateral root formation and root hair formation [50, 51]. Plant hormones are key elements for root morphology changes. Some beneficial bacteria are also described to produce them [51]. Regarding *B. subtilis* PTA-271 genome, it predicts the *trp* group, described in literature to produce tryptophan as the main precursor of the auxin IAA (indole-3-acetic acid) [42]. The genome of *B. subtilis* PTA-271 also predicts genes such as *yvdD* (Supplementary Table S2), linked in the literature to cytokinin synthesis which is known as a plant growth regulator (cell division, organogenesis) in combination with IAA. Gibberellins (GA) produced by some bacteria also affect the plant growth and survival [51]. Regarding the *B. subtilis* PTA-271 genome, it predicts *ispD* and *GerC3_HepT*, described in the literature to be respectively linked to 2-C-methyl-D-*erythritol* 4-phosphate (MEP) and geranylgeranyl diphosphate (GGPP) production, two successive precursors of GA and abscisic acid (ABA) synthesis in plants [52].

Genes described to encode for other plant growth regulators, namely polyamines (PAs), are also predicted in the genome of *B. subtilis* PTA-271. Among them: *speA*, *speB*, *speG* and *speE* are respectively described in literature to encode for putative ADC (arginine decarboxylase), agmatinase (leading to putrescine), then spermidine- and spermine- synthases. Additionally, genes encoding for putative S-adenosyl-methionine (SAM) decarboxylase (*speH*) and SAM-methyltransferase (S19-40_00450) are predicted in *B. subtilis* PTA-271 genome, and these proteins are mentioned to complete PA synthesis from putrescine [53]. PAs are known to promote flowering and to play important roles in inducing cell division, promoting regeneration of plant tissues and cell cultures [54], as delaying senescence [55].

Volatile compounds (VOCs) produced by some beneficial rhizospheric bacteria have also been identified as elicitors promoting plant growth. Regarding *B. subtilis* PTA-271, its predicted genes encode putatively for (1) acetoin (*acuA*, *acuC*…) and (2) 2,3-butanediol (*butA* and *butC*) [20, 56]. VOCs are especially reported to interact with plant hormones [57–59].

#### Host induced defenses and microbiota preservation: assets for plant protection

##### PLANT INDUCED DEFENSES upon biotic stress

Host primed defenses during ISR are regulated by hormones, depending on either JA and ET signaling or SA signaling [7, 8, 10, 60]. Beneficial microorganisms may modulate the plant hormonal balance or directly elicit the plant defenses. Regarding the genome of *B. subtilis* PTA-271, the *metK* gene is predicted to encode for SAM synthase that would appear ISR-useful for plants which possess the complementary ET metabolic machinery [53, 55]. SA is another hormone for which several genes encoding its metabolic pathways (from synthesis to hydrolysis) are predicted in *B. subtilis* PTA-271 genome, among which *pchA* putatively encoding for the salicylate biosynthesis isochorismate synthase.

Many elicitors also induce host immunity, coming from microorganisms (MAMPs, microbial associated molecular patterns) but also from the plant host (DAMPs, damage-associated molecular patterns). MAMPs can act from the external surface of a beneficial microorganism (flagellin) or result from a secretion outside or inside the host (surfactin, fengycin, VOCs, etc.) [58, 61–63]. Flagellin proteins are putatively encoded by the *hag* gene predicted in *B. subtilis* PTA-271 (Supplementary Table S1). The lipopeptides surfactin and fengycin are other elicitors of plant ISR putatively encoded by some genes predicted in the genome of *B. subtilis* PTA-271 (*srf* and *fen* genes, respectively). VOCs produced by rhizospheric bacteria, as the 3-hydroxy 2-butanone and acetoin which are putatively encoded by *B. subtilis* PTA-271 genome, are also well known to induce ISR [58]. Among VICs, the ubiquitous nitric oxide (NO) is another signal molecule [64]. Different genes related to NO metabolic pathways are predicted in *B. subtilis* PTA-271 genome, among which the gene *nos* putatively encoding for a NO synthase oxygenase. Exopolysaccharides (EPS) and lipopolysaccharides (LPS) are other elicitors reported in several *Bacillus* genera [9–14, 60]. Regarding the genome of *B. subtilis* PTA-271, it predicts several genes putatively encoding for EPS (S19-40_00800, S19-40_00870, S19-40_00999, S19-40_01009, S19-40_01427) and LPS (*lptB, lapA*, *lapB*), additionally to the other elicitors predicted to be encoded by *B. subtilis* PTA-271 genome (siderophores, flagella, N-acyl-L-homoserine lactone, etc.).

DAMPs are alternative elicitors produced by lytic enzymes (chitosan, glucans, etc.) of microorganisms (either beneficial or pathogenic) or plants [62]. Genes encoding for lytic enzymes are predicted in *B. subtilis* PTA-271 genome, such as those encoding for putative chitosanase and ß-glucanase (Supplementary Table S3). Many other genes are also predicted to encode for lytic enzymes in the *B. subtilis* PTA-271 spore cortex (Supplementary Table S4) for which the roles remain unclear.

##### PLANT INDUCED DEFENSES upon abiotic stress

Some previously cited hormones are also useful for plant defense against abiotic stress, such as ABA and GA [8], of which precursors are predicted to be encoded by genes identified in the genome of *B. subtilis* PTA-271 (*GerC3_HepT*, *ispD*). From GGPP, the kaurene pathway may lead to GA, while the phytoene path may lead to ABA [52], and in the genome of *B. subtilis* PTA-271, *yisP* (a *crtb* KEGG gene) encodes for a putative 15-cis-phytoene/all-trans-phytoene synthase. ET is another useful hormone for plant defense against abiotic stress [8], and *B. subtilis* PTA-271 genome has genes identified to putatively produce SAM (*metK*). Altogether these data predict that *B. subtilis* PTA-271 genome may putatively encode for key precursors of phytohormones that may influence actively ABA and ET contents in plants. In plants, ABA, GA and ET signaling pathways interfere altogether through different transcription factors (TF) or small proteins (GiD, DELLA, EIN, etc.) that physically interact [65, 66]. In the genome of *B. subtilis* PTA-271, many genes are predicted to encode for sigma factors and many TF (Supplementary Table S2). It is noteworthy to understand that useful TF upon abiotic stress could also be useful upon biotic stress. The set of genes under common regulatory controls (operons) are also listed in the Supplementary Table S2.

PAs such as those predicted to be encoded by the genome of *B. subtilis* PTA-271 are also described to protect plant cells upon water deficit [67], temperature changes [68] and salinity [69].

##### MICROBIOTA quality and preservation

As energy and carbon sources, plant root exudates (sugars, organic acids, amino acids, lipophilic compounds, etc.) would enable the selective recruitment of biosurfactant producers [70, 71]. In return, these beneficial bacteria can facilitate the bioavailability of root exudates and biofilm formation, thus the colonization of host-plants by beneficial bacteria [49, 70, 72], maybe such as *B. subtilis* PTA-271 which is suspected to produce surfactin. SA was also shown to mediate changes in the composition of root exudates, then in the qualitative microorganism recruitment by plants [19]. Regarding the *B. subtilis* PTA-271 genome, some genes are also predicted to produce SA (*pchA*), highlighting another key lever that putatively influence the composition of plant microbiome.

Beneficial microbial interactions can additionally depend on bacterial auto-inducers (AI) that are low-molecular weight signal molecules activating the interactive competences of a bacterium in a quorum-sensing (QS) dependent manner [73]. Among AI, the furanosyl-borate-diester (AI-2) is described as universal for interspecies communication both in Gram-positive and Gram-negative bacteria [74]. Regarding *B. subtilis* PTA-271 genome, the predicted *luxS* gene putatively encodes for AI-2 production, while the predicted *EntF* and *AM373* putatively encode oligopeptides or auto-inducing peptide (AIP) precursors. AIP is another class of AI consisting of 5–34 amino acids residues and produced by Gram-positive bacteria for their intercellular communication [75].

When interacting with the environment, a microorganism must also remain metabolically active to exert beneficial effects. Upon biotic interactions*, Bacillus* species are exposed to host defenses that include reactive oxygen species (ROS) [76]. Regarding the system of sensing, protection and regulation of ROS in the genome of *B. subtilis* PTA-271, genes are predicted to putatively encode for resistance to hydroperoxide (*ohrA, ohrB*, *ohrR*). Upon abiotic stress, beneficial bacteria must survive dehydration, wounding, cold, heat or salinity that in turn lead to regulation of the water status. For this end, bacterial species can control their intracellular solute pools [77, 78]. Regarding the genome of *B. subtilis* PTA-271, genes predicted to encode for potassium uptake proteins (*KtrA*, *KtrB*) putatively enable survival in high salinity environments. Interestingly, the genome of *B. subtilis* PTA-271 also predicts genes to detoxify or resist compounds accumulating in the environment [79, 80], such as arsenite (*arsR*), organic pesticides or nitroaromatic compounds (*sugE, qacC, mhqR, mhqA*) among others (Supplementary Tables S2 and S5).

Upon extreme environmental conditions, some beneficial bacteria can sporulate, turning on endospore form [1, 81]. Regarding the genome of *B. subtilis* PTA-271, several genes are predicted to be involved in the sporulation process (Supplementary Table S4): *spo* (sporulation control), *ger* (germination control), *cot* (endospore external layer) and *cw* (spore cortex lytic enzymes), putatively enabling it to survive long lasting periods while preserving all beneficial strengths for plant profits.

#### Direct confrontation with pathogens or aggressive molecules

Upon direct confrontation, *Bacillus* species also need to protect themselves against pathogen defenses. In addition to ROS protection, diverse transporters mediate antibiotic extrusion, whether specific to a substance or a group of substances. Regarding the genome of *B. subtilis* PTA-271, the specific transporters predicted would putatively confer it resistance towards: tetracyclin (*tetA*, *tetR*, *tetD*), fosfomycin (*fosB*), erythromycin (*msrA*, *msrB*), bacillibactin (*ymfD*), bacitracin (*BceA*, *BceB*, *BcrC*), bleomycin (*ble*) and riboflavin (*ribZ*, *rfnT*) for example. Among the non-specific transporters (or multidrug transporters) predicted in the genome of *B. subtilis* PTA-271 are: *mepA*, *ebrA* and *ebrB*; *ykkD* and *ykkC*; *bmrA* and *bmr3*; *emrY*, among others.

*Bacillus* species can additionally directly detoxify some pathogen aggressive molecules targeting plants, such as phytotoxins, by the mean of antitoxins or detoxifying enzymes such as transferases and CYP450s [82, 83]. In the genome of *B. subtilis* PTA-271, the main transferases predicted are glutathione-S-transferases GST, malonyl-transferases MT, glucosyl-transferases GT and many others, while the main CYP450s predicted are mono-oxygenases and dioxygenases (Supplementary Table S5). Quenching enzymes constitute another lever for beneficial bacteria to directly target pathogen aggressive molecules, by preventing their QS-dependent production [8, 84]. Indeed, *Bacillus* species share *aiiA* gene encoding for *N*-acetyl homoserine lactonase able to hydrolyze the lactone ring of the AHLs (Acyl-homoserine lactones) involved in the QS production of some pathogen virulent factors. The genome analysis of *B. subtilis* PTA-271 predicts such genes putatively encoding for quenching enzymes such as lactonases, β-lactamases, deaminases, deacetylases and other (de)acylases (Supplementary Table S6).

Polyketide synthases (PKS) are another type of transferases, namely acetyltransferases, described to produce plant beneficial molecules as microbicide for phytopathogens: the polyketides (PK) [85, 86]. Regarding the genome of *B. subtilis* PTA-271, 15 genes are predicted to encode for putative PKS, many others for acetyltransferases or for enzymes sharing similar part of the PKS functions (Supplementary Table S7). According to antiSMASH 5.1.0, *B. subtilis* PTA-271 genome predicts 11 secondary metabolites gene clusters, among which: 1 PKS cluster and 1 hybrid PKS-NRPS cluster (Supplementary Table S8).

An extensive range of pathogen direct effectors are additionally produced by *Bacillus* spp., such as the RP (ribosomally synthesized peptides) and NRP (non-ribosomally synthesized peptides) antimicrobial molecules [20, 87]. Some of them are predicted as encoded by the genome of *B. subtilis* PTA-271, such as: Baillaene (*pksD)*, subtilosin *(sboA, albG, albE, albD, albB, albA)* and bacilysin (*bacE, bacF, bacG*) (Supplementary Table S3). Lipopeptides are other NRP antimicrobial molecules [49, 88], which encoding genes are predicted in the genome of *B. subtilis* PTA-271 to putatively produce the powerful antifungal substances fengycin and surfactin (Supplementary Table S3). Besides antibiotics and surfactants, bacterial siderophores can also directly alter pathogen fitness and aggressiveness, by depriving pathogen growth of iron while providing it for plant growth [89]. Regarding the genome of *B. subtilis* PTA-271, predicted genes putatively encode for the siderophore Bacillibactin (Supplementary Table S3). Lytic enzymes (CWDE) are other important feature of *Bacillus* spp. that can both alter pathogen survival and produce MAMPs [90]. Regarding the genome of *B. subtilis* PTA-271, several genes are predicted to encode for putative CWDE: 1 chitosanase (*csn*), 1 β-glucanase (*bglS*), 1 β-glucanase / cellulase (*eglS*) and about 80 proteases (Supplementary Table S3).

Besides these NRP and RP antimicrobial molecules, the genome of *B. subtilis* PTA-271 also predicts the genes *hcnC*, *acu* and *but*, putatively encoding for the volatile antimicrobial compounds: VIC (hydrogen cyanide, HCN) and VOC (acetoin and 2,3-butanediol), respectively [8, 20, 56].

According to COG categories, 2.30% of *B. subtilis* PTA-271 genome is predicted to be devoted to the production of secondary metabolites, considered as one of the most important features in biocontrol activities. AntiSMASH 5.1.0 predicts 11 secondary metabolites gene clusters in *B. subtilis* PTA-271 genome, among which 3 NRPS clusters and 2 RiPPs clusters (Supplementary Table S8).

### *B. subtilis* PTA-271 GENOME COMPARISON WITH OTHER GENOMES

To understand the magnitude of the differences between *B. subtilis* PTA-271 and other *Bacillus* strains, the PTA-271 genome has been compared to the complete genomes of 5 type-strains (*B. subtilis* NCIB 3610, *B. subtilis* 168, *B. subtilis* 9407, *B. amyloliquefaciens subsp. plantarum* strain FZB42, and *B. velezensis* KTCT 13012) [91] and 32 non-type strains, represented in Table 6. Among non-type strains showing ≥99% of the *16S* ribosomal gene similarity with PTA-271 are 31 distinct strains of *B. subtilis* and 1 *Bacillus velezensis*. For this genomic comparison, was used the GGDC 2.1 web server [92], the DSMZ phylogenomics pipeline to estimate DNA-DNA hybridization (DDH) [92], and the JSpecies WS web server to estimate the Average Nucleotide Identity (ANI) through pairwise comparisons [93]. The DDH value was estimated using the recommended formula (formula two) for draft genomes, at the GGDC website [94]. The ANI values were calculated using Ezbiocloud [95]. The whole data analysis enabled to obtain the intergenomic distances between genomes and their probability of belonging to the same species or subspecies. The general comparison of genomes is reported in Table 6, while the intergenomic distances (DDH estimate and ANI) are shown in Table 7.

**Table 6:**
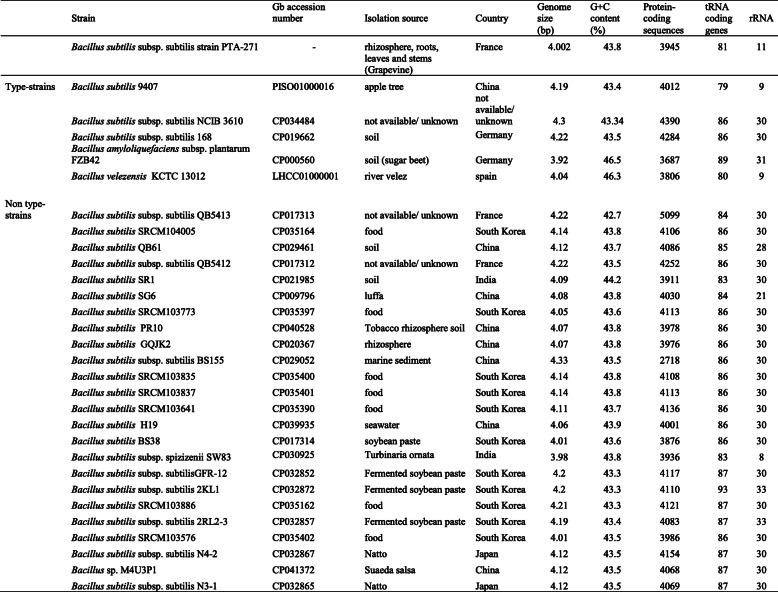
Comparative NCBI genome analysis of *Bacillus subtilis* PTA-271 with strains showing ≥99% of *16 s* similarity

**Table 7:**
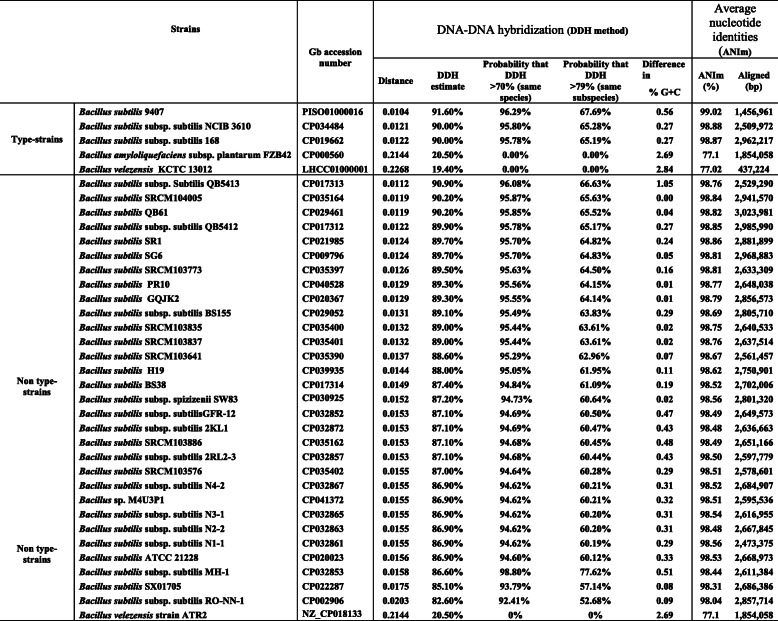
Comparative genome distances analysis with other strains*,* using DNA-DNA hybridization and average nucleotide identities

Among the type strain genomes, the closest strain to *B. subtilis* PTA-271 was *B. subtilis* 9407, with a 0.0104 distance, a DDH estimate of 91.60%, and an ANIm of 99.02%. As expected, the most distant strain was *B. velezensis* KTCT 13012, with a 0.2268 distance, a DDH estimate of 19.40% and a 0% probability of being the same species, corroborated with an ANIm percentage of 77.02%. Concerning the non-type strain genomes, the closer strains to PTA-271 were *B. subtilis* QB5413, *B. subtilis* SRCM 104005, and *B. subtilis* QB61 with distances of 0.0112, 0.0119 and 0.0119 respectively, and DDH estimates of 90.90, 90.20 and 90.20% respectively. The most distant strain was *B. velezensis* strain ATR2, with a distance of 0.2144 and a DDH estimate of 20.50% corroborated with an ANIm percentage of 77.1%. The most distant *B. subtilis* strain to PTA-271 was *B. subtilis* subsp. *subtilis* RO-NN-1 with a distance of 0.203 and a DDH of 82.60%.

## Conclusion

With a genome size of 4,001,755 bp containing 97.69% of protein encoding genes, the draft genome of *B. subtilis* PTA-271 highlights all the qualities of a promising plant beneficial microorganism. The most relevant predicted genes encode for: (1) a functional swarming motility system highlighting advantageous colonizing capacity of host and a strong interacting capacity within plant microbiota; (2) a strong survival capacity, due to sporulation but also to complex detoxifying systems, auto-inducing metabolic paths and recruiting capacities for adding microbiota values; and (3) the delivery of many bioactive substances (hormones, elicitors, effectors and quenchers, siderophores and lytic enzymes, etc.), facilitating the stimulation of plant growth or defenses, or else, disturbing pathogen fitness or aggressiveness. Interestingly, the putative capacity of *B. subtilis* PTA-271 to produce a wide range of phytohormone analogous (SA, ET precursor, ABA etc.), as well as diverse direct effectors and lytic enzymes against plant pathogens, highlight a significant potential for biocontrol strategies. Altogether, the plurality of the biomolecules putatively encoded by the genome of *B. subtilis* PTA-271 PTA-271 are putative strengths to impact both biochemical conditions, species ratios and their interactions, predicting an ability to combat a broad spectrum of plant pathogens such as grapevine trunk disease [3].

## Supplementary Information

**Additional file 1: Table S1.** *Bacillus subtilis* PTA-271 encoding genes for motility, adhesion and plant root colonizing capacity.

**Additional file 2: Table S2.** *Bacillus subtilis* PTA-271 encoding genes for some Transcriptional regulators and Operons.

**Additional file 3: Table S3.** *Bacillus subtilis* PTA-271 encoding genes for antimicrobial molecules, other effectors and lytic enzymes.

**Additional file 4: Table S4.** *Bacillus subtilis* PTA-271 encoding genes for sporulation.

**Additional file 5: Table S5.** *Bacillus subtilis* PTA-271 encoding genes for some CYP450 and for Transferases.

**Additional file 6: Table S6.** *Bacillus subtilis* PTA-271 encoding genes for lactonases, β-lactamases, deaminases, deacetylases.

**Additional file 7: Table S7.** *Bacillus subtilis* PTA-271 encoding genes for PKS and other acetyltransferases.

**Additional file 8: Table S8.** Anti-SMASH 5.1.0 prediction of gene clusters responsible for secondary metabolite production in *Bacillus subtilis* PTA-271.

## Data Availability

The whole genome shotgun project has been deposited at DDBJ/ENA/GenBank under the accession JACERQ000000000. The version described in this paper is version JACERQ000000000 and all related information is represented in Table 3.

## Abbreviations

ABA: abscisic acid
DAMPs: damage-associated molecular patterns
EPS: exopolysaccharides
ET: ethylene
GTD: grapevine trunk diseases
ISR: induced systemic resistance
JA: jasmonic acid
LPS: lipopolysaccharides
MAMPs: microbial associated molecular patterns
NO: nitric oxide
NRP: non-ribosomally synthesized peptides
PA: polyamines
PK: polyketides
RP: ribosomally synthesized antimicrobial peptides
RiPP: post-translationally modified RP
ROS: reactive oxygen species
SA: salicylic acid
ViC: inorganic volatile compound
VOC: organic volatile compound
