# Supplementary material for: Genome sequence analysis of the beneficial Bacillus subtilis PTA-271 isolated from a Vitis vinifera (cv. Chardonnay) rhizospheric soil: assets for sustainable biocontrol
Source: Environ Microbiome. 2021 Jan 29;16:3. doi: 10.1186/s40793-021-00372-3 (PMC8067347; doi:10.1186/s40793-021-00372-3)
Supplement: Supplementary file 1 — Additional file 1: Table S1. Bacillus subtilis PTA-271 encoding genes for motility, adhesion and plant root colonizing capacity. [file 40793_2021_372_MOESM1_ESM.pdf]

Table S1 : *Bacillus subtilis* PTA-271 encoding genes for motility, adhesion and plant root colonizing capacity

Locus tag ID	Gene	Function
	Motility (swarming), biofilm and root colonization	
S19-40_00277	*ylxH*	Flagellum site-determining protein
S19-40_00278	*flhF*	Flagellar biosynthesis protein
S19-40_00279	*flhA*	Flagellar biosynthesis protein
S19-40_00280	*flhB*	Flagellar biosynthetic protein
S19-40_00283	*fliP*	Flagellar biosynthetic protein
S19-40_00286	*fliN*	Flagellar motor switch protein
S19-40_00287	*fliM*	Flagellar motor switch protein
S19-40_00289	*flgG*	Flagellar basal-body rod protein
S19-40_00293	*fliJ*	Flagellar protein
S19-40_00296	*fliG*	Flagellar motor switch protein
S19-40_00297	*fliF*	Flagellar M-ring protein
S19-40_00298	*fliE*	Flagellar hook-basal body complex protein
S19-40_00299	*flgC*	Flagellar basal-body rod protein
S19-40_00300	*flgB*	Flagellar basal body rod protein
S19-40_00290	*flgD*	Basal-body rod modification protein
S19-40_00310	*flhB*	Flagellar biosynthetic protein
S19-40_00890	*flgK*	Flagellar hook-associated protein 1
S19-40_00891	*flaB3*	Flagellar filament 31 kDa core protein
S19-40_00893	*fliW*	Flagellar assembly factor
S19-40_00895	*hag*	Flagellin
S19-40_00897	*fliD*	B-type flagellar hook-associated protein 2
S19-40_00898	*fliS*	Flagellar protein
S19-40_00557	*motB*	Motility protein B
S19-40_02745	*cheR*	Chemotaxis protein methyltransferase
S19-40_00115	*cheY*	Chemotaxis protein
S19-40_00272	*cheD*	Chemoreceptor glutamine deamidase
S19-40_00273	*cheC*	CheY-P phosphatase
S19-40_00274	*cheW*	Chemotaxis protein
S19-40_00275	*cheA*	Chemotaxis protein
S19-40_00276	*cheB*	Chemotaxis response regulator protein-glutamate methylesterase
S19-40_00285	*cheY*	Chemotaxis protein
S19-40_00519	*cheV*	Chemotaxis protein
S19-40_02745	*cheR*	Chemotaxis protein methyltransferase
S19-40_03165	*swrC*	Swarming motility protein
S19-40_00882	*spo0A*	Response regulator receiver domain protein
