# Supplementary material for: Genome sequence analysis of the beneficial Bacillus subtilis PTA-271 isolated from a Vitis vinifera (cv. Chardonnay) rhizospheric soil: assets for sustainable biocontrol
Source: Environ Microbiome. 2021 Jan 29;16:3. doi: 10.1186/s40793-021-00372-3 (PMC8067347; doi:10.1186/s40793-021-00372-3)
Supplement: Supplementary file 2 — Additional file 2: Table S2. Bacillus subtilis PTA-271 encoding genes for some Transcriptional regulators and Operons. [file 40793_2021_372_MOESM2_ESM.pdf]

Table S2 : *Bacillus subtilis* PTA-271 encoding genes for some Transcriptional regulators and Operons

Locus tag ID	Gene	Function
		Transcriptional regulators
S19-40_00006	purR	LacI family transcriptional regulator, purine nucleotide synthesis repressor
S19-40_00012	immR	HTH-type transcriptional regulator ImmR
S19-40_00023	gltC	HTH-type transcriptional regulator GltC
S19-40_00025	sinR	XRE family transcriptional regulator, master regulator for biofilm formation
S19-40_00049	slyA	Transcriptional regulator SlyA
S19-40_00060	gltC	HTH-type transcriptional regulator GltC
S19-40_00064	yofA	HTH-type transcriptional regulator YofA
S19-40_00173	glnR	HTH-type transcriptional regulator GlnR
S19-40_00212	betI	HTH-type transcriptional regulator BetI
S19-40_00255	infB	Translation initiation factor IF-2
S19-40_00268	tsf	Elongation factor Ts
S19-40_00374	dksA	RNA polymerase-binding transcription factor DksA
S19-40_00374	yocK	General stress protein 16O
S19-40_00405	mraZ	Transcriptional regulator MraZ
S19-40_00506	argP	HTH-type transcriptional regulator ArgP
S19-40_00516	ykuD	Putative L,D-transpeptidase YkuD
S19-40_00529	splA	Transcriptional regulator protein (SplA)
S19-40_00549	ytcD	putative HTH-type transcriptional regulator YtcD
S19-40_00558	mhqR	HTH-type transcriptional regulator MhqR
S19-40_00593	mhqR	HTH-type transcriptional regulator MhqR
S19-40_00596	tnrA	HTH-type transcriptional regulator TnrA
S19-40_00599	splA	Transcriptional regulator protein (SplA)
S19-40_00614	ohrR	Organic hydroperoxide resistance transcriptional regulator
S19-40_00667	yusO	putative HTH-type transcriptional regulator YusO
S19-40_00690	crp	CRP/FNR family transcriptional regulator, cyclic AMP receptor protein
S19-40_00758	ywnA	Putative HTH-type transcriptional regulator YwnA
S19-40_00761	zntR	Zn(II)-responsive transcriptional regulator
S19-40_00768	hipB	Transcriptional regulator, y4mF family
S19-40_00779	-	putative HTH-type transcriptional regulator/GBAA_1941/BAS1801
S19-40_00794	ulaR	HTH-type transcriptional regulator UlaR
S19-40_00809	gltR	HTH-type transcriptional regulator GltR
S19-40_00814	lrpC	HTH-type transcriptional regulator LrpC
S19-40_00823	hdfR	HTH-type transcriptional regulator HdfR
S19-40_00834	scrR	LacI family transcriptional regulator, sucrose operon repressor
S19-40_00841	ywtF	Putative transcriptional regulator YwtF
S19-40_00865	lytR	Transcriptional regulator LytR
S19-40_00879	yvhJ	Putative transcriptional regulator YvhJ
S19-40_00882	degU	Transcriptional regulatory protein DegU
S19-40_00882	*spo0A*	Response regulator receiver domain protein
S19-40_00912	betI	HTH-type transcriptional regulator BetI
S19-40_00924	zitR	Transcriptional regulator ZitR
S19-40_00927	adcR	Transcriptional regulator AdcR
S19-40_00929	nanR	Transcriptional regulator NanR
S19-40_00962	phoB	Phosphate regulon transcriptional regulatory protein PhoB
S19-40_00987	yvdT	putative HTH-type transcriptional regulator YvdT
S19-40_00997	sinR	HTH-type transcriptional regulator SinR
S19-40_01016	lutR	HTH-type transcriptional regulator LutR
S19-40_01017	lacR	HTH-type transcriptional regulator LacR
S19-40_01028	desR	Transcriptional regulatory protein DesR
S19-40_01035	hdfR	HTH-type transcriptional regulator HdfR
S19-40_01050	opcR	HTH-type transcriptional regulator, osmoprotectant uptake regulator
S19-40_01060	opcR	HTH-type transcriptional regulator, osmoprotectant uptake regulator
S19-40_01067	yodB	HTH-type transcriptional regulator YodB
S19-40_01068	rghR	HTH-type transcriptional repressor RghR
S19-40_01069	rghR	HTH-type transcriptional repressor RghR
S19-40_01078	tetR	Mycofactocin system transcriptional regulator
S19-40_01084	csoR	Copper-sensing transcriptional repressor CsoR
S19-40_01118	walR	Transcriptional regulatory protein WalR
S19-40_01132	liaR	Transcriptional regulatory protein LiaR
S19-40_01138	ethR	HTH-type transcriptional regulator EthR
S19-40_01140	cssR	Transcriptional regulatory protein CssR
S19-40_01148	gltR	HTH-type transcriptional regulator GltR
S19-40_01152	yusO	putative HTH-type transcriptional regulator YusO
S19-40_01185	yurK	putative HTH-type transcriptional regulator YurK
S19-40_01273	comA	Transcriptional regulatory protein ComA
S19-40_01288	dcuR	Transcriptional regulatory protein DcuR
S19-40_01325	ulaR	HTH-type transcriptional regulator UlaR
S19-40_01340	opcR	HTH-type transcriptional repressor OpcR

S19-40_01355	-	Transcriptional regulator, Acidobacterial, PadR-family
S19-40_01359	tetR	Tetracycline repressor protein class B from transposon Tn10
S19-40_01367	csoR	Copper-sensing transcriptional repressor CsoR
S19-40_01369	tipA	HTH-type transcriptional activator TipA
S19-40_01375	gltR	HTH-type transcriptional regulator GltR
S19-40_01379	gltR	HTH-type transcriptional regulator GltR
S19-40_01398	cmpR	HTH-type transcriptional activator CmpR
S19-40_01412	adhR	HTH-type transcriptional regulator AdhR
S19-40_01421	dgaR	Transcriptional regulatory protein DagR
S19-40_01424	-	Sugar-specific transcriptional regulator TrmB
S19-40_01430	bm3R1	HTH-type transcriptional repressor Bm3R1
S19-40_01468	cymR	HTH-type transcriptional regulator CymR
S19-40_01482	comN	Post-transcriptional regulator ComN
S19-40_01499	yebC	putative transcriptional regulatory protein YebC
S19-40_01559	yusO	putative HTH-type transcriptional regulator YusO
S19-40_01574	betI	HTH-type transcriptional regulator BetI
S19-40_01606	infC	Translation initiation factor IF-3
S19-40_01618	nrdR	Transcriptional repressor NrdR
S19-40_01621	ytcD	putative HTH-type transcriptional regulator YtcD
S19-40_01629	phoP	Alkaline phosphatase synthesis transcriptional regulatory protein PhoP
S19-40_01660	cmpR	HTH-type transcriptional activator CmpR
S19-40_01683	yttP	putative HTH-type transcriptional regulator YttP
S19-40_01722	ytzE	putative HTH-type transcriptional regulator YtzE
S19-40_01733	yesS	HTH-type transcriptional regulator YesS
S19-40_01744	lacR	HTH-type transcriptional regulator LacR
S19-40_01764	ytrA	HTH-type transcriptional repressor YtrA
S19-40_01802	dksA	RNA polymerase-binding transcription factor DksA
S19-40_01845	gmuR	HTH-type transcriptional regulator GmuR
S19-40_01860	rspR	HTH-type transcriptional repressor RspR
S19-40_01863	slyA	Transcriptional regulator SlyA
S19-40_01866	tcaR	HTH-type transcriptional regulator TcaR
S19-40_01872	betI	HTH-type transcriptional regulator BetI
S19-40_01894	cmtR	HTH-type transcriptional regulator CmtR
S19-40_01896	norG	HTH-type transcriptional regulator NorG
S19-40_01900	aseR	HTH-type transcriptional repressor AseR
S19-40_01901	-	putative HTH-type transcriptional regulator
S19-40_01904	yybR	putative HTH-type transcriptional regulator YybR
S19-40_01906	yybR	putative HTH-type transcriptional regulator YybR
S19-40_01909	gabR	HTH-type transcriptional regulatory protein GabR
S19-40_01921	rhaS	HTH-type transcriptional activator RhaS
S19-40_01923	carD	RNA polymerase-binding transcription factor CarD
S19-40_01927	-	Transcriptional regulator, y4mF family
S19-40_01969	dcuR	Transcriptional regulatory protein DcuR
S19-40_01990	lrpC	HTH-type transcriptional regulator LrpC
S19-40_01999	mtlR	Transcriptional regulator MtlR
S19-40_02005	kipR	HTH-type transcriptional regulator KipR
S19-40_02028	gabR	HTH-type transcriptional regulatory protein GabR
S19-40_02044	srrA	Transcriptional regulatory protein SrrA
S19-40_02058	benM	HTH-type transcriptional regulator BenM
S19-40_02064	gabR	HTH-type transcriptional regulatory protein GabR
S19-40_02082	hpr	HTH-type transcriptional regulator Hpr
S19-40_02087	yciB	Putative L,D-transpeptidase YciB
S19-40_02114	mhqR	HTH-type transcriptional regulator MhqR
S19-40_02148	natR	Transcriptional regulatory protein NatR
S19-40_02154	yxaF	putative HTH-type transcriptional regulator YxaF
S19-40_02167	walR	Transcriptional regulatory protein WalR
S19-40_02172	lutR	HTH-type transcriptional regulator LutR
S19-40_02207	-	Transcriptional regulator SlyA
S19-40_02257	ybbH	putative HTH-type transcriptional regulator YbbH
S19-40_02291	xre	HTH-type transcriptional regulator Xre
S19-40_02305	exuR	putative HTH-type transcriptional repressor ExuR
S19-40_02344	manR	Transcriptional regulator ManR
S19-40_02402	spxA	Transcriptional regulator, Spx/MgsR family
S19-40_02449	-	Transcriptional regulator, Acidobacterial, PadR-family
S19-40_02468	norG	HTH-type transcriptional regulator NorG
S19-40_02472	degA	HTH-type transcriptional regulator DegA
S19-40_02497	sgrR	HTH-type transcriptional regulator SgrR
S19-40_02539	bm3R1	HTH-type transcriptional repressor Bm3R1
S19-40_02555	hpr	HTH-type transcriptional regulator Hpr
S19-40_02602	gabR	HTH-type transcriptional regulatory protein GabR
S19-40_02607	cysL	HTH-type transcriptional regulator CysL
S19-40_02612	nsrR	HTH-type transcriptional repressor NsrR

S19-40_02617	liaR	Transcriptional regulatory protein LiaR
S19-40_02643	ytrA	HTH-type transcriptional repressor YtrA
S19-40_02786	srrA	Transcriptional regulatory protein SrrA
S19-40_02834	xre	HTH-type transcriptional regulator Xre
S19-40_02872	ykuD	Putative L,D-transpeptidase YkuD
S19-40_02880	-	Zn(II)-responsive transcriptional regulator
S19-40_02904	argR	Arginine repressor
S19-40_02924	efp	Elongation factor P
S19-40_02931	mntR	Transcriptional regulator MntR
S19-40_02940	sinR	HTH-type transcriptional regulator SinR
S19-40_02959	spxA	Transcriptional regulator, Spx/MgsR family
S19-40_02992	zur	Fur family transcriptional regulator, zinc uptake regulator
S19-40_03061	marR	MarR family transcriptional regulator, multiple antibiotic resistance protein MarR
S19-40_03147	lrpC	HTH-type transcriptional regulator LrpC
S19-40_03152	trpR	TrpR family transcriptional regulator, trp operon repressor
S19-40_03164	kstR2	HTH-type transcriptional repressor KstR2
S19-40_03183	-	putative transcriptional regulatory protein
S19-40_03199	yesS	HTH-type transcriptional regulator YesS
S19-40_03219	mprA	Transcriptional repressor MprA
S19-40_03221	IF5B	translation initiation factor 5B
S19-40_03237	yfmP	HTH-type transcriptional regulator YfmP
S19-40_03254	citT	Transcriptional regulatory protein CitT
S19-40_03272	treR	HTH-type transcriptional regulator TreR
S19-40_03308	glvR	HTH-type transcriptional regulator GlvR
S19-40_03316	degU	Transcriptional regulatory protein DegU
S19-40_03325	yusO	putative HTH-type transcriptional regulator YusO
S19-40_03379	-	Transcriptional regulator PadR-like family protein
S19-40_03402	liaR	Transcriptional regulatory protein LiaR
S19-40_03450	mgrA	HTH-type transcriptional regulator MgrA
S19-40_03463	cynR	HTH-type transcriptional regulator CynR
S19-40_03472	slrA	Transcriptional regulator SlrA
S19-40_03472	slrA	Transcriptional regulator SlrA
S19-40_03494	-	Transcriptional regulator, Acidobacterial, PadR-family
S19-40_03530	cysL	HTH-type transcriptional regulator CysL
S19-40_03537	iscR	HTH-type transcriptional regulator IscR
S19-40_03547	btr	HTH-type transcriptional activator Btr
S19-40_03554	czrA	HTH-type transcriptional repressor CzrA
S19-40_03562	desR	Transcriptional regulatory protein DesR
S19-40_03567	dksA	RNA polymerase-binding transcription factor DksA
S19-40_03599	yodB	HTH-type transcriptional regulator YodB
S19-40_03634	yusO	putative HTH-type transcriptional regulator YusO
S19-40_03677	kdgR	HTH-type transcriptional regulator KdgR
S19-40_03703	tcrX	putative transcriptional regulatory protein TcrX
S19-40_03711	walR	Transcriptional regulatory protein WalR
S19-40_03729	yybR	putative HTH-type transcriptional regulator YybR
S19-40_03734	mta	HTH-type transcriptional activator mta
S19-40_03739	gltC	HTH-type transcriptional regulator GltC
S19-40_03743	slyA	Transcriptional regulator SlyA
S19-40_03753	-	putative HTH-type transcriptional regulator
S19-40_03760	purR	HTH-type transcriptional repressor PurR
S19-40_03790	yydK	putative HTH-type transcriptional regulator YydK
S19-40_03799	gntR	putative D-xylose utilization operon transcriptional repressor
S19-40_03804	nicR	MarR family transcriptional regulator, lower aerobic nicotinate degradation pathway regulator
S19-40_03806	yxaF	putative HTH-type transcriptional regulator YxaF
S19-40_03815	dhaS	HTH-type dhaKLM operon transcriptional activator DhaS
S19-40_03885	infA	Translation initiation factor IF-1
S19-40_03911	tuf	Elongation factor Tu
S19-40_03912	fusA	Elongation factor G
S19-40_03940	ctsR	Transcriptional regulator CtsR
S19-40_03944	-	Transcriptional regulator, y4mF family
		Operons
S19-40_00023	cysL	LysR family transcriptional regulator, transcriptional activator of the cysJI operon
S19-40_00049	HpaR	homoprotocatechuate degradation operon regulator, HpaR
S19-40_00060	gltC	LysR family transcriptional regulator, transcription activator of glutamate synthase operon
S19-40_00271	fliA	RNA polymerase sigma factor for flagellar operon FliA
S19-40_00371	pyrR	pyrimidine operon attenuation protein / uracil phosphoribosyltransferase
S19-40_00482	fruR2, fruR	DeoR family transcriptional regulator, fructose operon transcriptional repressor
S19-40_00506	cysL	LysR family transcriptional regulator, transcriptional activator of the cysJI operon
S19-40_00529	splA	transcriptional regulator of the spore photoproduct lyase operon
S19-40_00533	glcT	PtsGHI operon antiterminator
S19-40_00558	HpaR	HpaR: homoprotocatechuate degradation operon regulator, HpaR
S19-40_00593	HpaR	HpaR: homoprotocatechuate degradation operon regulator, HpaR

S19-40_00599	splA	transcriptional regulator of the spore photoproduct lyase operon
S19-40_00614	HpaR	HpaR: homoprotocatechuate degradation operon regulator, HpaR
S19-40_00779	HpaR	HpaR: homoprotocatechuate degradation operon regulator, HpaR
S19-40_00823	hcaR	Hca operon transcriptional activator HcaR
S19-40_00834	scrR	LacI family transcriptional regulator, sucrose operon repressor
S19-40_00884	comFA	ComF operon protein 1
S19-40_00885	comFB	ComF operon protein 2
S19-40_00887	YvyF	YvyF: flagellar operon protein
S19-40_00912	mexL	TetR/AcrR family transcriptional regulator, mexJK operon transcriptional repressor
S19-40_00927	HpaR	HpaR: homoprotocatechuate degradation operon regulator, HpaR
S19-40_01035	gltC	LysR family transcriptional regulator, transcription activator of glutamate synthase operon
S19-40_01037	araR	GntR family transcriptional regulator, arabinose operon transcriptional repressor
S19-40_01078	acrR	TetR/AcrR family transcriptional regulator, multidrug resistance operon repressor
S19-40_01106	btr	AraC family transcriptional regulator, transcriptional activator for feuABC-ybbA operon
S19-40_01116	fliA	RNA polymerase sigma factor for flagellar operon FliA
S19-40_01138	nemR	TetR/AcrR family transcriptional regulator, transcriptional repressor for nem operon
S19-40_01152	HpaR	HpaR: homoprotocatechuate degradation operon regulator, HpaR
S19-40_01185	frlR	GntR family transcriptional regulator, frlABCD operon transcriptional regulator
S19-40_01325	srlR	Glucitol operon repressor
S19-40_01365	pspE	phageshock_pspE: phage shock operon rhodanese PspE
S19-40_01421	gfrR	sigma-54 dependent transcriptional regulator, gfr operon transcriptional activator
S19-40_01430	fatR, bscR	TetR/AcrR family transcriptional regulator, repressor of fatR-cypB operon
S19-40_01559	HpaR	HpaR: homoprotocatechuate degradation operon regulator, HpaR
S19-40_01660	ytlI	LysR family transcriptional regulator, regulator of the ytmI operon
S19-40_01683	fatR, bscR	TetR/AcrR family transcriptional regulator, repressor of fatR-cypB operon
S19-40_01695	cytR	LacI family transcriptional regulator, repressor for deo operon, udp, cdd, tsx, nupC, and nupG
S19-40_01733	btr	AraC family transcriptional regulator, transcriptional activator for feuABC-ybbA operon
S19-40_01744	cytR	LacI family transcriptional regulator, repressor for deo operon, udp, cdd, tsx, nupC, and nupG
S19-40_01764	-	trehalos_R_Bsub: trehalose operon repressor
S19-40_01771	caiE	Carnitine operon protein CaiE
S19-40_01845	-	trehalos_R_Bsub: trehalose operon repressor
S19-40_01860	rspR	GntR family transcriptional regulator, rspAB operon transcriptional repressor
S19-40_01901	mexL	TetR/AcrR family transcriptional regulator, mexJK operon transcriptional repressor
S19-40_01911	fatR, bscR	TetR/AcrR family transcriptional regulator, repressor of fatR-cypB operon
S19-40_01921	rhaS	AraC family transcriptional regulator, L-rhamnose operon regulatory protein RhaS
S19-40_01984	bcsB	cellulose synthase operon protein B
S19-40_01999	mtlR	mannitol operon transcriptional antiterminator
S19-40_02105	gltC	LysR family transcriptional regulator, transcription activator of glutamate synthase operon
S19-40_02114	HpaR	HpaR: homoprotocatechuate degradation operon regulator, HpaR
S19-40_02154	lmrA, yxaF	TetR/AcrR family transcriptional regulator, lmrAB and yxaGH operons repressor
S19-40_02186	-	trehalos_R_Bsub: trehalose operon repressor
S19-40_02202	rhaS	AraC family transcriptional regulator, L-rhamnose operon regulatory protein RhaS
S19-40_02233	arsR	Arsenical resistance operon repressor
S19-40_02262	btr	AraC family transcriptional regulator, transcriptional activator for feuABC-ybbA operon
S19-40_02263	btr	AraC family transcriptional regulator, transcriptional activator for feuABC-ybbA operon
S19-40_02344	manR	activator of the mannose operon, transcriptional antiterminator
S19-40_02472	cytR	LacI family transcriptional regulator, repressor for deo operon, udp, cdd, tsx, nupC, and nupG
S19-40_02473	araC	Arabinose operon regulatory protein
S19-40_02500	cytR	LacI family transcriptional regulator, repressor for deo operon, udp, cdd, tsx, nupC, and nupG
S19-40_02539	fatR, bscR	TetR/AcrR family transcriptional regulator, repressor of fatR-cypB operon
S19-40_02594	merR1	Mercuric resistance operon regulatory protein
S19-40_02623	glpP	Glycerol uptake operon antiterminator regulatory protein
S19-40_02649	HpaR	HpaR: homoprotocatechuate degradation operon regulator, HpaR
S19-40_02717	birA	BirA family transcriptional regulator, biotin operon repressor / biotin---[acetyl-CoA-carboxylase] ligase
S19-40_02933	pspE	phageshock_pspE: phage shock operon rhodanese PspE
S19-40_02946	comGG	ComG operon protein 7
S19-40_02950	comGC	ComG operon protein 3
S19-40_02952	comGA	ComG operon protein 1
S19-40_03041	comEC	ComE operon protein 3
S19-40_03042	comEB	ComE operon protein 2
S19-40_03043	comEA	ComE operon protein 1
S19-40_03075	nemR	TetR/AcrR family transcriptional regulator, transcriptional repressor for nem operon
S19-40_03104	gutR	LuxR family transcriptional regulator, glucitol operon activator
S19-40_03152	trpR	TrpR family transcriptional regulator, trp operon repressor
S19-40_03199	btr	AraC family transcriptional regulator, transcriptional activator for feuABC-ybbA operon
S19-40_03219	emrR, mprA	MarR family transcriptional regulator, negative regulator of the multidrug operon emrRAB
S19-40_03237	yfmP	MerR family transcriptional regulator, repressor of the yfmOP operon
S19-40_03272	-	trehalos_R_Bsub: trehalose operon repressor
S19-40_03300	acoR	Acetoin dehydrogenase operon transcriptional activator AcoR
S19-40_03308	glvR	RpiR family transcriptional regulator, glv operon transcriptional regulator
S19-40_03325	HpaR	HpaR: homoprotocatechuate degradation operon regulator, HpaR
S19-40_03357	perR	Peroxide operon regulator

S19-40_03384	licT, bglG	beta-glucoside operon transcriptional antiterminator
S19-40_03432	licR	putative licABCH operon regulator
S19-40_03450	HpaR	HpaR: homoprotocatechuate degradation operon regulator, HpaR
S19-40_03453	sacY	Levansucrase and sucrase synthesis operon antiterminator
S19-40_03463	gltC	LysR family transcriptional regulator, transcription activator of glutamate synthase operon
S19-40_03473	acrR, smeT	TetR/AcrR family transcriptional regulator, acrAB operon repressor
S19-40_03488	sacY	Levansucrase and sucrase synthesis operon antiterminator
S19-40_03530	cysL	LysR family transcriptional regulator, transcriptional activator of the cysJI operon
S19-40_03547	btr	AraC family transcriptional regulator, transcriptional activator for feuABC-ybbA operon
S19-40_03634	HpaR	HpaR: homoprotocatechuate degradation operon regulator, HpaR
S19-40_03642	acoR	sigma-54 dependent transcriptional regulator, acetoin dehydrogenase operon transcriptional activator AcoR
S19-40_03677	kdgR	LacI family transcriptional regulator, kdg operon repressor
S19-40_03739	gltC	LysR family transcriptional regulator, transcription activator of glutamate synthase operon
S19-40_03760	scrR	LacI family transcriptional regulator, sucrose operon repressor
S19-40_03790	-	trehalos_R_Bsub: trehalose operon repressor
S19-40_03799	gntR	putative D-xylose utilization operon transcriptional repressor
S19-40_03804	HpaR	HpaR: homoprotocatechuate degradation operon regulator, HpaR
S19-40_03806	lmrA, yxaF	TetR/AcrR family transcriptional regulator, lmrAB and yxaGH operons repressor
S19-40_03815	dhaS	HTH-type dhaKLM operon transcriptional activator DhaS
S19-40_03823	srlR	Glucitol operon repressor
S19-40_03839	btr	AraC family transcriptional regulator, transcriptional activator for feuABC-ybbA operon
S19-40_03856	lsrR	lsr operon transcriptional repressor
S19-40_03865	hutP	Hut operon positive regulatory protein
S19-40_03979	purR	Pur operon repressor
