# Supplementary material for: Genome sequence analysis of the beneficial Bacillus subtilis PTA-271 isolated from a Vitis vinifera (cv. Chardonnay) rhizospheric soil: assets for sustainable biocontrol
Source: Environ Microbiome. 2021 Jan 29;16:3. doi: 10.1186/s40793-021-00372-3 (PMC8067347; doi:10.1186/s40793-021-00372-3)
Supplement: Supplementary file 3 — Additional file 3: Table S3. Bacillus subtilis PTA-271 encoding genes for antimicrobial molecules, other effectors and lytic enzymes. [file 40793_2021_372_MOESM3_ESM.pdf]

Table S3 : *Bacillus subtilis* PTA-271 encoding genes for antimicrobial molecules, other effectors and lytic enzymes

Locus tag ID	Gene	Function
		Bacteriocin/Bacteriocin-like-peptides
S19-40_00081	menE	AMP-binding enzyme
S19-40_00234	albE	Antilisterial bacteriocin subtilosin biosynthesis protein AlbE
S19-40_00679	albG	Antilisterial bacteriocin subtilosin biosynthesis protein AlbG
S19-40_00681	albE	Antilisterial bacteriocin subtilosin biosynthesis protein AlbE
S19-40_00682	albD	Antilisterial bacteriocin subtilosin biosynthesis protein AlbD
S19-40_00683	btuD	Lantibiotic protection ABC transporter, ATP-binding subunit
S19-40_00684	albB	Antilisterial bacteriocin subtilosin biosynthesis protein AlbB
S19-40_00685	albA	Antilisterial bacteriocin subtilosin biosynthesis protein AlbA
S19-40_00690	crp	CRP/FNR family transcriptional regulator, cyclic AMP receptor protein
S19-40_00726	ywlC	Threonylcarbamoyl-AMP synthase
S19-40_00946	hisI	Phosphoribosyl-AMP cyclohydrolase
S19-40_00964	btuD	Putative bacteriocin export ABC transporter, lactococcin 972 group
S19-40_01244	dhbE	2,3-dihydroxybenzoate-AMP ligase
S19-40_01246	-	AMP-binding enzyme
S19-40_01575	-	AMP-binding enzyme
S19-40_01689	-	AMP-binding enzyme
S19-40_01755	bceB	Bacitracin export permease protein BceB
S19-40_01756	bceA	Bacitracin export ATP-binding protein BceA
S19-40_01915	-	Bacteriocin-protection, YdeI or OmpD-Associated
S19-40_01966	nukF, mcdF, sboF	lantibiotic transport system ATP-binding protein
S19-40_01998	-	AMP-binding enzyme
S19-40_01998	-	AMP-binding enzyme
S19-40_02046	btuD	Putative bacteriocin export ABC transporter, lactococcin 972 group
S19-40_02071	licA	lichenysin synthetase A
S19-40_02165	btuD	lantibiotic protection ABC transporter, ATP-binding subunit
S19-40_02198	-	Trypsin
S19-40_02227	skfF	Putative bacteriocin-SkfA transport system permease protein SkfF
S19-40_02641	btuD	Lantibiotic protection ABC transporter, ATP-binding subunit
S19-40_03432	licR	lichenan operon transcriptional antiterminator
S19-40_03433	licB	Lichenan-specific phosphotransferase enzyme IIB component
S19-40_03434	licC	Lichenan permease IIC component
S19-40_03435	licA	Lichenan-specific phosphotransferase enzyme IIA component
S19-40_03456	licC	Lichenan permease IIC component
S19-40_03706	-	Trypsin-like peptidase domain protein
S19-40_03836	btuD	Putative bacteriocin export ABC transporter, lactococcin 972 group
		Other effectors
S19-40_00073	fenC	fengycin family lipopeptide synthetase A
S19-40_00074	fenD	fengycin family lipopeptide synthetase B
S19-40_00075	-	fengycin family lipopeptide synthetase C
S19-40_00076	fenA	fengycin family lipopeptide synthetase D
S19-40_00077	fenB	fengycin family lipopeptide synthetase E
S19-40_00208	pksD	bacillaene synthase trans-acting acyltransferase
S19-40_00567	bacF	Transaminase BacF
S19-40_00679	albG	Antilisterial bacteriocin subtilosin biosynthesis protein AlbG
S19-40_00681	albE	Antilisterial bacteriocin subtilosin biosynthesis protein AlbE
S19-40_00682	albD	Antilisterial bacteriocin subtilosin biosynthesis protein AlbD
S19-40_00684	albB	Antilisterial bacteriocin subtilosin biosynthesis protein AlbB

S19-40_00684	albB	Antilisterial bacteriocin subtilosin biosynthesis protein AlbB
S19-40_00685	albA	Antilisterial bacteriocin subtilosin biosynthesis protein AlbA
S19-40_00686	sboA	Subtilosin-A
S19-40_02068	srfAD	Surfactin synthase thioesterase subunit
S19-40_02069	srfAC	Surfactin synthase subunit 3
S19-40_02070	srfAB	Surfactin synthase subunit 2
S19-40_02071	srfAA	Surfactin synthase subunit 1
S19-40_03525	bacE	Putative bacilysin exporter BacE
S19-40_03526	bacF	Transaminase BacF
S19-40_03527	bacG	NADPH-dependent reductase BacG
	Siderophores	
S19-40_01242	dhbA	2,3-dihydro-2,3-dihydroxybenzoate dehydrogenase
S19-40_01243	dhbC	Isochorismate synthase DhbC
S19-40_01244	dhbE	2,3-dihydroxybenzoate-AMP ligase
S19-40_01245	dhbB	Isochorismatase
S19-40_01246	dhbF	Dimodular nonribosomal peptide synthase
	Lytic enzymes	
S19-40_00094	-	Cellulase (glycosyl hydrolase family 5)
S19-40_00541	sleB	Spore cortex-lytic enzyme
S19-40_00651	sleB	spore cortex-lytic enzyme
S19-40_01400	csn	Chitosanase
S19-40_02204	sleB	spore cortex-lytic enzyme
S19-40_02296	sleB	spore cortex-lytic enzyme
S19-40_02768	sleB	Spore cortex-lytic enzyme
S19-40_03055	sleB	spore cortex-lytic enzyme
S19-40_03077	sleB	spore cortex-lytic enzyme
S19-40_03385	bglS	Beta-glucanase
	Proteases	
S19-40_00020	ydeA	putative protease YdeA
S19-40_00164	-	CAAX protease self-immunity
S19-40_00193	aprX	Serine protease AprX
S19-40_00233	albF	Putative zinc protease AlbF
S19-40_00239	clpP	ATP-dependent Clp protease proteolytic subunit
S19-40_00247	-	putative zinc protease
S19-40_00262	rasP	Regulator of sigma-W protease RasP
S19-40_00302	clpY	ATP-dependent protease ATPase subunit ClpY
S19-40_00303	clpQ	ATP-dependent protease subunit ClpQ
S19-40_00414	lon	Lon protease
S19-40_00475	-	Thermophilic metalloprotease (M29)
S19-40_00555	clpE	ATP-dependent Clp protease ATP-binding subunit ClpE
S19-40_00578	htpX	Protease HtpX
S19-40_00609	isp	Major intracellular serine protease
S19-40_00640	htrA	Serine protease Do-like HtrA
S19-40_00669	rip2	Putative zinc metalloprotease Rip2
S19-40_00680	albF	Putative zinc protease AlbF
S19-40_00908	ctpB	Carboxy-terminal processing protease CtpB
S19-40_00980	clpP	ATP-dependent Clp protease proteolytic subunit
S19-40_01141	htrB	Serine protease Do-like HtrB
S19-40_01229	paiB	Protease synthase and sporulation protein PAI 2
S19-40_01403	lasA	LasA protease
S19-40_01404	lasA	LasA protease
S19-40_01415	yraA	Putative cysteine protease YraA
S19-40_01448	ydcP	putative protease YdcP

S19-40_01449	ydcP	putative protease YdcP
S19-40_01512	-	Cysteine protease Prp
S19-40_01538	lon1	Lon protease 1
S19-40_01539	lon2	Lon protease 2
S19-40_01540	clpX	ATP-dependent Clp protease ATP-binding subunit ClpX
S19-40_01953	gluP	Rhomboid protease GluP
S19-40_02198	mpr	Extracellular metalloprotease
S19-40_02280	-	Putative phage serine protease XkdF
S19-40_02300	ftsH	ATP-dependent zinc metalloprotease FtsH
S19-40_02446	nprB	Neutral protease B
S19-40_02479	wprA	Cell wall-associated protease
S19-40_02527	htpX	Protease HtpX
S19-40_02732	bepA	Beta-barrel assembly-enhancing protease
S19-40_02769	prsW	Protease PrsW
S19-40_02776	-	CAAX protease self-immunity
S19-40_02969	gluP	Rhomboid protease GluP
S19-40_03037	gpr	Germination protease
S19-40_03091	tsaD	Glycoprotease family protein
S19-40_03093	tsaD	Glycoprotease family protein
S19-40_03100	-	CAAX protease self-immunity
S19-40_03218	yccA	modulator of FtsH protease
S19-40_03275	pfpL	Intracellular protease, PfpI family
S19-40_03413	htpX	Protease HtpX
S19-40_03455	epr	Minor extracellular protease Epr
S19-40_03486	vpr	Minor extracellular protease vpr
S19-40_03605	ctpA	Carboxy-terminal processing protease CtpA
S19-40_03706	htrA	Serine protease Do-like HtrA
S19-40_03756	-	CAAX protease self-immunity
S19-40_03937	clpX	ATP-dependent Clp protease ATP-binding subunit ClpX
S19-40_03955	ftsH	ATP-dependent zinc metalloprotease FtsH
S19-40_03982	yabG	Sporulation-specific protease YabG
