# Supplementary material for: Genome sequence analysis of the beneficial Bacillus subtilis PTA-271 isolated from a Vitis vinifera (cv. Chardonnay) rhizospheric soil: assets for sustainable biocontrol
Source: Environ Microbiome. 2021 Jan 29;16:3. doi: 10.1186/s40793-021-00372-3 (PMC8067347; doi:10.1186/s40793-021-00372-3)
Supplement: Supplementary file 4 — Additional file 4: Table S4. Bacillus subtilis PTA-271 encoding genes for sporulation. [file 40793_2021_372_MOESM4_ESM.pdf]

Table S4 : *Bacillus subtilis* PTA-271 encoding genes for sporulation

Locus tag ID	Gene	Function
	Sporolation related genes	
S19-40_00078	*yjcA*	Sporulation protein YjcA
S19-40_00117	*ynzD*	Spo0E like sporulation regulatory protein
S19-40_00119	*sirA*	Sporulation inhibitor of replication protein SirA
S19-40_00176	*spoVK*	Stage V sporulation protein K
S19-40_00177	*cwlC*	Sporulation-specific N-acetylmuramoyl-L-alanine amidase
S19-40_00222	*spoVS*	Stage V sporulation protein S
S19-40_00246	*ylmC*	Sporulation protein, YlmC/YmxH family
S19-40_00387	*spoIIGA*	Sporulation sigma-E factor-processing peptidase
S19-40_00401	*spoVD*	Stage V sporulation protein D
S19-40_00416	*ylbJ*	Sporulation integral membrane protein YlbJ
S19-40_00471	*kinA*	Sporulation kinase A
S19-40_00495	-	Sporulation protein cse15
S19-40_00522	*kinA*	Sporulation kinase A
S19-40_00539	*stoA*	Sporulation thiol-disulfide oxidoreductase A
S19-40_00540	*ykvU*	Sporulation protein YkvU
S19-40_00541	*sleB*	Spore cortex-lytic enzyme
S19-40_00545	*ydhD*	Putative sporulation-specific glycosylase YdhD
S19-40_00546	*ykvP*	Spore protein YkvP
S19-40_00547	*ykvP*	Spore protein YkvP
S19-40_00559	*kinD*	Sporulation kinase D
S19-40_00561	*spo0E*	stage 0 sporulation regulatory protein
S19-40_00573	*kinE*	Sporulation kinase E
S19-40_00649	*spoIISA*	Stage II sporulation protein SA
S19-40_00650	*spoIISB*	Stage II sporulation protein SB
S19-40_00708	*spo0F*	Sporulation initiation phosphotransferase F
S19-40_00746	*spoIID*	stage II sporulation protein D
S19-40_00766	*spoIIQ*	Stage II sporulation protein Q
S19-40_00780	*spoIIID*	Stage III sporulation protein D
S19-40_00844	*gerBB*	Spore germination protein B2
S19-40_00845	*gerBA*	Spore germination protein B1
S19-40_00958	*whiA*	Sporulation transcription regulator WhiA
S19-40_00982	*cotR*	Putative sporulation hydrolase CotR
S19-40_01032	*ydhD*	Putative sporulation-specific glycosylase YdhD
S19-40_01057	*sdpC*	Sporulation delaying protein C
S19-40_01058	*sdpB*	Sporulation-delaying protein SdpB
S19-40_01059	*sdpA*	Sporulation-delaying protein SdpA
S19-40_01133	*gerAC*	Spore germination protein A3
S19-40_01134	*gerAB*	Spore germination protein A2
S19-40_01135	*gerAA*	Spore germination protein A1
S19-40_01208	*yunB*	Sporulation protein YunB
S19-40_01211	*YhcN*	Sporulation lipoprotein, YhcN/YlaJ family
S19-40_01229	*paiB*	Protease synthase and sporulation protein PAI 2
S19-40_01261	*Ytvl*	Sporulation integral membrane protein YtvI
S19-40_01297	*kinE*	Sporulation kinase E
S19-40_01298	*kinE*	Sporulation kinase E
S19-40_01483	*spoVB*	Stage V sporulation protein B

S19-40_01500	*coxA*	Sporulation cortex protein CoxA
S19-40_01501	*safA*	SpoIVD-associated factor A
S19-40_01510	*spo0B*	Sporulation initiation phosphotransferase B
S19-40_01514	*spoIVFB*	Stage IV sporulation protein FB
S19-40_01515	*spoIVFA*	Stage IV sporulation protein FA
S19-40_01523	*spoIIB*	Stage II sporulation protein B
S19-40_01529	*spoVID*	Stage VI sporulation protein D
S19-40_01557	*gerM*	Spore germination protein GerM
S19-40_01560	*gerE*	Spore germination protein GerE
S19-40_01642	*ytrH*	Sporulation membrane protein YtrH
S19-40_01643	*ytrI*	Sporulation membrane protein YtrI
S19-40_01727	-	Sporulation protein cse60
S19-40_01859	*ydhD*	Putative sporulation-specific glycosylase YdhD
S19-40_01951	*ydcC*	Sporulation protein YdcC
S19-40_02076	*nucB*	Sporulation-specific extracellular nuclease
S19-40_02162	*cwlJ*	Spore cortex-lytic enzyme
S19-40_02177	*spo0A*	Stage 0 sporulation protein A
S19-40_02216	-	Sigma-G-dependent sporulation-specific SASP protein
S19-40_02229	*skfC*	Sporulation-killing factor biosynthesis protein SkfC
S19-40_02230	*skfB*	Sporulation killing factor maturation protein SkfB
S19-40_02231	*skfA*	Sporulation killing factor
S19-40_02296	*cwlA*	Spore cortex-lytic enzyme
S19-40_02371	*yjcA*	Sporulation protein YjcA
S19-40_02372	*cotV*	Spore coat protein V
S19-40_02373	*cotW*	Spore coat protein W
S19-40_02374	*cotX*	Spore coat protein X
S19-40_02375	*cotY*	Spore coat protein Y
S19-40_02376	*cotZ*	Spore coat protein Z
S19-40_02450	*sdpA*	Sporulation-delaying protein SdpA
S19-40_02451	*sdpB*	Sporulation-delaying protein SdpB
S19-40_02452	*sdpC*	Sporulation delaying protein C
S19-40_02483	*yisI*	Spo0E like sporulation regulatory protein
S19-40_02484	*gerPA*	Spore germination protein gerPA/gerPF
S19-40_02485	*gerPB*	Spore germination GerPB
S19-40_02486	*gerPC*	Spore germination protein GerPC
S19-40_02487	*gerPD*	Spore germination protein GerPD
S19-40_02488	*gerPE*	Spore germination protein GerPE
S19-40_02489	*gerPF*	Spore germination protein gerPA/gerPF
S19-40_02559	*yhaL*	Sporulation protein YhaL
S19-40_02674	*spo0M*	Sporulation-control protein spo0M
S19-40_02725	*YpjB*	Sporulation protein YpjB
S19-40_02753	*spoIVA*	Stage IV sporulation protein A
S19-40_02767	*ypeB*	Sporulation protein YpeB
S19-40_02768	*sleB*	Spore cortex-lytic enzyme
S19-40_02812	*spoVAF*	stage V sporulation protein AF
S19-40_02813	-	stage V sporulation protein AE
S19-40_02814	-	stage V sporulation protein AE
S19-40_02815	*spoVAD*	stage V sporulation protein AD
S19-40_02816	*spoVAC*	stage V sporulation protein AC
S19-40_02817	*spoVAB*	stage V sporulation protein AB

S19-40_02818	*spoVAA*	stage V sporulation protein AA
S19-40_02820	*spoIIAB*	stage II sporulation protein AB (anti-sigma F factor)
S19-40_02821	*spoIIAA*	stage II sporulation protein AA (anti-sigma F factor antagonist)
S19-40_02828	*spoIIM*	Stage II sporulation protein M
S19-40_02901	*spo0A*	Stage 0 sporulation protein A
S19-40_02902	*spoIVB*	SpoIVB peptidase
S19-40_02915	*spoIIIAH*	Stage III sporulation protein AH
S19-40_02918	*spoIIIAE*	Stage III sporulation protein AE
S19-40_03018	*yqfD*	Sporulation protein YqfD
S19-40_03019	*yqfC*	Sporulation protein YqfC
S19-40_03055	*cwlH*	Spore cortex-lytic enzyme
S19-40_03059	*nucB*	Sporulation-specific extracellular nuclease
S19-40_03771	*soj*	Sporulation initiation inhibitor protein Soj
S19-40_03960	*spoIIE*	Stage II sporulation protein E
S19-40_03969	*gerD*	Stage V sporulation protein B
S19-40_03970	*spoVT*	Stage V sporulation protein T
S19-40_03977	*spoVG*	Putative septation protein SpoVG
S19-40_03982	*yabG*	Sporulation-specific protease YabG
S19-40_04013	*yaaH*	Spore germination protein YaaH
