# Supplementary material for: Genome sequence analysis of the beneficial Bacillus subtilis PTA-271 isolated from a Vitis vinifera (cv. Chardonnay) rhizospheric soil: assets for sustainable biocontrol
Source: Environ Microbiome. 2021 Jan 29;16:3. doi: 10.1186/s40793-021-00372-3 (PMC8067347; doi:10.1186/s40793-021-00372-3)
Supplement: Supplementary file 5 — Additional file 5: Table S5. Bacillus subtilis PTA-271 encoding genes for some CYP450 and for Transferases. [file 40793_2021_372_MOESM5_ESM.pdf]

Table S5 : *Bacillus subtilis* PTA-271 encoding genes for some CYP450 and for Transferases.

Locus tag ID	Gene	Function
		P450 mono-oxigenases
S19-40_00039	hpaB	4-hydroxyphenylacetate 3-monooxygenase
S19-40_01036	luxA	Alkanal monooxygenase alpha chain
S19-40_01390	ncd2/npd	Nitronate monooxygenase
S19-40_01651	moxC	Putative monooxygenase MoxC
S19-40_01653	camP	2,5-diketocamphane 1,2-monooxygenase
S19-40_02002	mhuD, hmoB	Antibiotic biosynthesis monooxygenase
S19-40_02030	ycnE	Putative monooxygenase YcnE
S19-40_02134	limB	Limonene 1,2-monooxygenase
S19-40_02347	-	Antibiotic biosynthesis monooxygenase
S19-40_02506	otcC	Anhydrotetracycline monooxygenase
S19-40_02544	hmoB	Heme-degrading monooxygenase HmoB
S19-40_02664	ssuD	Alkanesulfonate monooxygenase
S19-40_02844	-	Antibiotic biosynthesis monooxygenase
S19-40_03213	hmoA	Heme-degrading monooxygenase HmoA
S19-40_03220	kmo	Kynurenine 3-monooxygenase
S19-40_03485	luxB	Alkanal monooxygenase beta chain
S19-40_03847	ntaA	Nitrilotriacetate monooxygenase component A
		dioxygenases
S19-40_00125	fosB	Glyoxalase/Bleomycin resistance protein/Dioxygenase superfamily protein
S19-40_00563	mtnD	Acireductone dioxygenase
S19-40_00645	mhqA	Putative ring-cleaving dioxygenase MhqA
S19-40_00719	-	Glyoxalase/Bleomycin resistance protein/Dioxygenase superfamily protein
S19-40_01175	-	Glyoxalase/Bleomycin resistance protein/Dioxygenase superfamily protein
S19-40_01333	cdoA	Cysteine dioxygenase
S19-40_01883	mhqO	Putative ring-cleaving dioxygenase MhqO
S19-40_02093	hcaC	3-phenylpropionate/cinnamic acid dioxygenase ferredoxin subunit
S19-40_02313	hcaD	3-phenylpropionate/cinnamic acid dioxygenase ferredoxin--NAD(+) reductase component
S19-40_02851	-	Glyoxalase/Bleomycin resistance protein/Dioxygenase superfamily protein
S19-40_02870	-	Glyoxalase/Bleomycin resistance protein/Dioxygenase superfamily protein
S19-40_03214	-	Glyoxalase/Bleomycin resistance protein/Dioxygenase superfamily protein
S19-40_03313	catE	Catechol-2,3-dioxygenase
S19-40_03371	-	2OG-Fe dioxygenase
S19-40_03457	-	Glyoxalase/Bleomycin resistance protein/Dioxygenase superfamily protein
S19-40_03602	mhqE	Putative ring-cleaving dioxygenase MhqE
S19-40_03717	-	Glyoxalase/Bleomycin resistance protein/Dioxygenase superfamily protein
S19-40_03759	-	Glyoxalase/Bleomycin resistance protein/Dioxygenase superfamily protein
S19-40_03807	qdoI	Quercetin 2,3-dioxygenase
		GST-GT-MT related genes
S19-40_00083	gcvH	Glycine cleavage system H protein
S19-40_00328	fabD	Malonyl CoA-acyl carrier protein transacylase
S19-40_00397	ftsW	putative peptidoglycan glycosyltransferase FtsW
S19-40_00399	mraY	Phospho-N-acetylmuramoyl-pentapeptide-transferase
S19-40_00434	ftsW	putative peptidoglycan glycosyltransferase FtsW
S19-40_00547	-	Glycosyl transferases group 1
S19-40_00588	-	Glycosyl transferase family 2
S19-40_00592	ugtP	Processive diacylglycerol beta-glucosyltransferase
S19-40_00642	-	putative glycosyltransferase
S19-40_00852	-	glyco_rSAM_CFB: glycosyltransferase, GG-Bacteroidales peptide system
S19-40_00853	-	Glycosyl transferase WecB/TagA/CpsF family protein
S19-40_00856	-	Glycosyl transferase family 2
S19-40_00857	-	CDP-Glycerol:Poly(glycerophosphate) glycerophosphotransferase
S19-40_00861	-	Glycosyl transferase 1 domain A
S19-40_00862	-	Glycosyl transferases group 1
S19-40_00863	-	Glycosyl transferase family 2
S19-40_00869	-	Glycosyl transferase family 2
S19-40_00872	-	Glycosyl transferases group 1
S19-40_00876	-	Glycosyl transferase family 2
S19-40_00877	-	Glycosyl transferases group 1
S19-40_00878	-	Glycosyl transferase family 4
S19-40_01001	-	Glycosyl transferases group 1
S19-40_01002	-	Glycosyl transferase family 2
S19-40_01003	-	Glycosyl transferases group 1
S19-40_01005	-	Glycosyl transferase family 2

S19-40_01007	-	Glycosyl transferase family 2
S19-40_01589	-	Glyoxalase-like domain protein
S19-40_01806	-	Glycosyl transferases group 1
S19-40_01809	-	Glycosyl transferases group 1
S19-40_01858	-	UDP-glucoronosyl and UDP-glucosyl transferase
S19-40_01985	-	Glycosyltransferase like family 2
S19-40_01997	-	Methyltransferase domain protein
S19-40_02321	-	MGT: glycosyltransferase, MGT family
S19-40_02705	-	Transglycosylase
S19-40_02719	-	Glycosyl transferases group 1
S19-40_02740	-	Glycosyl transferase family, a/b domain
S19-40_02973	-	Glycosyl transferases group 1
S19-40_03228	-	Glycosyl transferase family 2
S19-40_03344	-	Glycosyl transferase family 2
S19-40_03391	-	Methyltransferase domain protein
S19-40_03452	-	Glycosyl transferase family 8
S19-40_03483	-	Cell cycle protein
S19-40_03497	-	Glycosyl transferase family 2
S19-40_03504	-	Glycosyl transferase family 2
S19-40_03586	-	MGT: glycosyltransferase, MGT family
S19-40_03626	-	Glycosyl transferase family 2
S19-40_03656	-	Monogalactosyldiacylglycerol (MGDG) synthase
S19-40_03859	-	Glycosyl transferase family, a/b domain
		Other transferases
S19-40_00001	araP	L-arabinose transport system permease protein AraP
S19-40_00002	araQ	L-arabinose transport system permease protein AraQ
S19-40_00053	ydaF	Putative ribosomal N-acetyltransferase YdaF
S19-40_00065	-	g_glut_trans: gamma-glutamyltransferase
S19-40_00083	-	methylmalonyl-CoA carboxyltransferase 1.3S subunit
S19-40_00089	gtaB	UTP--glucose-1-phosphate uridylyltransferase
S19-40_00090	DPM1	dolichol-phosphate mannosyltransferase
S19-40_00101	plsY	Glycerol-3-phosphate acyltransferase
S19-40_00174	pat	Putative phenylalanine aminotransferase
S19-40_00185	miaA	tRNA dimethylallyltransferase
S19-40_00207	-	Acyl transferase domain protein
S19-40_00208	-	Acyl transferase domain protein
S19-40_00219	miaB	miaB-methiolase: tRNA-i(6)A37 thiotransferase enzyme MiaB
S19-40_00456	arnB	UDP-4-amino-4-deoxy-L-arabinose--oxoglutarate aminotransferase
S19-40_00588	arnC	Undecaprenyl-phosphate 4-deoxy-4-formamido-L-arabinose transferase
S19-40_00642	arnC	Undecaprenyl-phosphate 4-deoxy-4-formamido-L-arabinose transferase
S19-40_00842	-	MFS transporter, SP family, arabinose:H+ symporter
S19-40_00856	arnC	Undecaprenyl-phosphate 4-deoxy-4-formamido-L-arabinose transferase
S19-40_00869	arnC	Undecaprenyl-phosphate 4-deoxy-4-formamido-L-arabinose transferase
S19-40_00876	arnC	Undecaprenyl-phosphate 4-deoxy-4-formamido-L-arabinose transferase
S19-40_01002	arnC	Undecaprenyl-phosphate 4-deoxy-4-formamido-L-arabinose transferase
S19-40_01005	arnC	Undecaprenyl-phosphate 4-deoxy-4-formamido-L-arabinose transferase
S19-40_01007	arnC	Undecaprenyl-phosphate 4-deoxy-4-formamido-L-arabinose transferase
S19-40_01011	arnB	UDP-4-amino-4-deoxy-L-arabinose--oxoglutarate aminotransferase
S19-40_01037	araR	Arabinose metabolism transcriptional repressor
S19-40_01038	araE	Arabinose-proton symporter
S19-40_01182	araP	L-arabinose transport system permease protein AraP
S19-40_01183	araQ	L-arabinose transport system permease protein AraQ
S19-40_01592	araQ	L-arabinose transport system permease protein AraQ
S19-40_01593	araP	L-arabinose transport system permease protein AraP
S19-40_01599	araA	L-arabinose isomerase
S19-40_01735	araQ	L-arabinose transport system permease protein AraQ
S19-40_01999	mtlR	mannitol operon transcriptional antiterminator
S19-40_02017	mtlD	Mannitol-1-phosphate 5-dehydrogenase
S19-40_02018	mtlF	Mannitol-specific phosphotransferase enzyme IIA component
S19-40_02019	mtlA	PTS system mannitol-specific EIICB component
S19-40_02074	kdsD, kpsF	arabinose-5-phosphate isomerase
S19-40_02473	araC	Arabinose operon regulatory protein
S19-40_02501	arnB	UDP-4-amino-4-deoxy-L-arabinose--oxoglutarate aminotransferase
S19-40_02720	mshB	thiol_BshB1: bacillithiol biosynthesis deacetylase BshB1
S19-40_02770	ypdA	Bthiol_YpdA: putative bacillithiol system oxidoreductase, YpdA family
S19-40_03197	araQ	L-arabinose transport system permease protein AraQ
S19-40_03210	araQ	L-arabinose transport system permease protein AraQ

S19-40_03228	arnC	Undecaprenyl-phosphate 4-deoxy-4-formamido-L-arabinose transferase
S19-40_03323	yfiT	DinB superfamily protein
S19-40_03344	arnC	Undecaprenyl-phosphate 4-deoxy-4-formamido-L-arabinose transferase
S19-40_03497	arnC	Undecaprenyl-phosphate 4-deoxy-4-formamido-L-arabinose transferase
S19-40_03506	arnB	UDP-4-amino-4-deoxy-L-arabinose--oxoglutarate aminotransferase
S19-40_03591	BshB2	thiol_BshB2: bacillithiol biosynthesis deacetylase BshB2
S19-40_03695	mshD	Mycothiol acetyltransferase
S19-40_03700	rocD	Ornithine aminotransferase
S19-40_03723	arnT, pmrK	4-amino-4-deoxy-L-arabinose transferase
S19-40_03723	-	Dolichyl-phosphate-mannose-protein mannosyltransferase
S19-40_03744	-	Acetyltransferase
S19-40_03746	wecD	dTDP-fucosamine acetyltransferase
S19-40_03758	maa	Maltose O-acetyltransferase
S19-40_03774	rsmG	Ribosomal RNA small subunit methyltransferase G
S19-40_03791	-	phosphotransferase system, EIIB
S19-40_03848	ytmI	putative N-acetyltransferase YtmI
S19-40_03859	-	Glycosyl transferase family, a/b domain
S19-40_03911	cysN	Sulfate adenylyltransferase subunit 1
S19-40_03918	rlmG	Ribosomal RNA large subunit methyltransferase G
S19-40_03927	-	Putative TrmH family tRNA/rRNA methyltransferase
S19-40_03930	cysE	Serine acetyltransferase
S19-40_03933	ispD	2-C-methyl-D-erythritol 4-phosphate cytidylyltransferase
S19-40_03948	-	ilvE_I: branched-chain amino acid aminotransferase
S19-40_03976	glmU	glmU: UDP-N-acetylglucosamine diphosphorylase/glucosamine-1-phosphate N-acetyltransferase
S19-40_03983	rsmA	Ribosomal RNA small subunit methyltransferase A
S19-40_03989	rsmI	Ribosomal RNA small subunit methyltransferase I
S19-40_03991	yfiC	tRNA1(Val) (adenine(37)-N6)-methyltransferase
S19-40_03998	glyA	Serine hydroxymethyltransferase
