# Supplementary material for: Genome sequence analysis of the beneficial Bacillus subtilis PTA-271 isolated from a Vitis vinifera (cv. Chardonnay) rhizospheric soil: assets for sustainable biocontrol
Source: Environ Microbiome. 2021 Jan 29;16:3. doi: 10.1186/s40793-021-00372-3 (PMC8067347; doi:10.1186/s40793-021-00372-3)
Supplement: Supplementary file 6 — Additional file 6: Table S6. Bacillus subtilis PTA-271 encoding genes for lactonases, β-lactamases, deaminases, deacetylases. [file 40793_2021_372_MOESM6_ESM.pdf]

Table S6 : *Bacillus subtilis* PTA-271 encoding genes for lactonases, β-lactamases, deaminases, deacetylases

Locus tag ID	Gene	Function
	lactone hydrolase	
S19-40_01709	ahlD, aiiA, attM, blcC	N-acyl homoserine lactone hydrolase
S19-40_02014	-	Dienelactone hydrolase family protein
S19-40_02855	ahlD, aiiA, attM, blcC	N-acyl homoserine lactone hydrolase
S19-40_03601	-	Dienelactone hydrolase family protein
	β-lactamases	
S19-40_00019	penP	Beta-lactamase
S19-40_00225	-	Beta-lactamase
S19-40_00240	-	Metallo-beta-lactamase superfamily protein
S19-40_00467	-	Metallo-beta-lactamase superfamily protein
S19-40_00991	-	Beta-lactamase
S19-40_01661	-	Beta-lactamase superfamily domain protein
S19-40_01709	-	Metallo-beta-lactamase superfamily protein
S19-40_02214	ybxI	putative beta-lactamase YbxI
S19-40_02259	-	Beta-lactamase
S19-40_02532	-	Beta-lactamase superfamily domain protein
S19-40_02855	-	Metallo-beta-lactamase superfamily protein
S19-40_02861	-	Beta-lactamase superfamily domain protein
S19-40_02961	-	Metallo-beta-lactamase superfamily protein
S19-40_03257	-	Metallo-beta-lactamase superfamily protein
S19-40_03550	bla2, blm, ccrA, blaB	metallo-beta-lactamase class B
S19-40_03707	-	Metallo-beta-lactamase superfamily protein
S19-40_03742	bla2, blm, ccrA, blaB	metallo-beta-lactamase class B
	deaminases	
S19-40_00468	adeC	Adenine deaminase
S19-40_00611	guaD	Guanine deaminase
S19-40_00930	nagB	Glucosamine-6-phosphate deaminase 1
S19-40_01533	hemC	Porphobilinogen deaminase
S19-40_01675	mtaD	5-methylthioadenosine/S-adenosylhomocysteine deaminase
S19-40_02187	nagB	Glucosamine-6-phosphate deaminase 1
S19-40_02802	tadA	tRNA-specific adenosine deaminase
S19-40_03012	cdd	Cytidine deaminase
S19-40_03042	tadA	tRNA-specific adenosine deaminase
S19-40_03150	yerA	Putative adenine deaminase YerA
S19-40_03978	yabJ	2-iminobutanoate/2-iminopropanoate deaminase
S19-40_04015	tadA	tRNA-specific adenosine deaminase
	deacetylases	
S19-40_00248	pdaA	Peptidoglycan-N-acetylmuramic acid deacetylase PdaA
S19-40_00383	argE	Acetylornithine deacetylase
S19-40_00501	ykuR	N-acetyldiaminopimelate deacetylase
S19-40_00931	nagA	N-acetylglucosamine-6-phosphate deacetylase
S19-40_01649	-	N-acetyldiaminopimelate deacetylase
S19-40_01692	-	Histone deacetylase domain protein
S19-40_02104	cah	Cephalosporin-C deacetylase
S19-40_02121	-	N-acetyldiaminopimelate deacetylase
S19-40_02333	pdaC	Peptidoglycan-N-acetylmuramic acid deacetylase PdaC
S19-40_02535	lysK	N-acetyl-lysine deacetylase
S19-40_02547	-	N-acetyldiaminopimelate deacetylase
S19-40_02585	pgdA	Peptidoglycan-N-acetylglucosamine deacetylase
S19-40_02586	cobB	NAD-dependent protein deacetylase
S19-40_02720	bshB1	N-acetyl-alpha-D-glucosaminyl L-malate deacetylase 1
S19-40_02857	argE	acetylornithine deacetylase
S19-40_03287	pdaA	Peptidoglycan-N-acetylmuramic acid deacetylase PdaA
S19-40_03411	icaB	Poly-beta-1,6-N-acetyl-D-glucosamine N-deacetylase
S19-40_03591	bshB2	putative N-acetyl-alpha-D-glucosaminyl L-malate deacetylase 2
S19-40_03620	argE	Acetylornithine deacetylase
S19-40_03852	-	N-acetyldiaminopimelate deacetylase
S19-40_03867	pdaA	Peptidoglycan-N-acetylmuramic acid deacetylase PdaA
	other deacetylases	
S19-40_02720	-	GlcNAc-PI de-N-acetylase
S19-40_03287	-	spore_pdaA: delta-lactam-biosynthetic de-N-acetylase
S19-40_03591	-	GlcNAc-PI de-N-acetylase
S19-40_02720	-	GlcNAc-PI de-N-acetylase
S19-40_03287	-	spore_pdaA: delta-lactam-biosynthetic de-N-acetylase
S19-40_03591	-	GlcNAc-PI de-N-acetylase
S19-40_03824	mmsA, iolA, ALDH6A1	malonate-semialdehyde dehydrogenase (acetylating) / methylmalonate-semialdehyde dehydrogenase
	other (de)acylases	

S19-40_00208	fabD	Malonyl CoA-acyl carrier protein transacylase
S19-40_00210	pksC	Polyketide biosynthesis malonyl CoA-acyl carrier protein transacylase PksC
S19-40_00328	fabD	Malonyl CoA-acyl carrier protein transacylase
S19-40_01475	dtd	D-aminoacyl-tRNA deacylase
S19-40_02338	ybaK	Cys-tRNA(Pro)/Cys-tRNA(Cys) deacylase YbaK
S19-40_02881	DBT, bkdB	2-oxoisovalerate dehydrogenase E2 component (dihydrolipoyl transacylase)
S19-40_03846	-	Penicillin acylase
