# Supplementary material for: Genome sequence analysis of the beneficial Bacillus subtilis PTA-271 isolated from a Vitis vinifera (cv. Chardonnay) rhizospheric soil: assets for sustainable biocontrol
Source: Environ Microbiome. 2021 Jan 29;16:3. doi: 10.1186/s40793-021-00372-3 (PMC8067347; doi:10.1186/s40793-021-00372-3)
Supplement: Supplementary file 7 — Additional file 7: Table S7. Bacillus subtilis PTA-271 encoding genes for PKS and other acetyltransferases. [file 40793_2021_372_MOESM7_ESM.pdf]

Table S7 : *Bacillus subtilis* PTA-271 encoding genes for PKS and other acetyltransferases

Locus tag ID	Gene	Function
Polyketide related genes		
S19-40_00196	pksS	Polyketide biosynthesis cytochrome P450 PksS
S19-40_00197	pksR	Polyketide synthase PksR
S19-40_00198	pksN	Polyketide synthase PksN
S19-40_00199	pksM	Polyketide synthase PksM
S19-40_00200	pksL	Polyketide synthase PksL
S19-40_00201	pksJ	Polyketide synthase PksJ
S19-40_00202	pksI	Putative polyketide biosynthesis enoyl-CoA isomerase PksI
S19-40_00203	pksH	putative polyketide biosynthesis enoyl-CoA hydratase PksH
S19-40_00204	pksG	Polyketide biosynthesis 3-hydroxy-3-methylglutaryl-ACP synthase PksG
S19-40_00205	pksF	Polyketide biosynthesis malonyl-ACP decarboxylase PksF
S19-40_00206	acpK	Polyketide biosynthesis acyl-carrier-protein AcpK
S19-40_00207	pksE	Polyketide biosynthesis protein PksE
S19-40_00208	pksD	Polyketide biosynthesis acyltransferase PksD
S19-40_00210	pksC	Polyketide biosynthesis malonyl CoA-acyl carrier protein transacylase PksC
S19-40_00211	pksB	putative polyketide biosynthesis zinc-dependent hydrolase PksB
S19-40_01385	pksS	Polyketide biosynthesis cytochrome P450 PksS
Acyl transferase related genes		
S19-40_00207	pfaD	Acyl transferase domain protein
S19-40_00208	fabD	Malonyl CoA-acyl carrier protein transacylase
S19-40_00210	fabD	Acyl transferase domain protein
S19-40_00210	pksC	Polyketide biosynthesis malonyl CoA-acyl carrier protein transacylase PksC
S19-40_00328	fabD	Malonyl CoA-acyl carrier protein transacylase
S19-40_00328	fabD	Malonyl CoA-acyl carrier protein transacylase
S19-40_02881	DBT, bkdB	2-oxoisovalerate dehydrogenase E2 component (dihydrolipoyl transacylase)
S19-40_03846	-	Penicillin acylase
Keto related genes		
S19-40_00120	tkt	Transketolase
S19-40_00205	fabF	beta-ketoacyl-acyl-carrier-protein synthase II
S19-40_00220	-	8-amino-7-oxononanoate synthase/2-amino-3-ketobutyrate coenzyme A ligase
S19-40_00407	apbA	Ketopantoate reductase PanE/ApbA
S19-40_00460	dxs	Transketolase, pyrimidine binding domain
S19-40_00476	: 2-dehydropantoate	Ketopantoate reductase PanE/ApbA
S19-40_00565	mtnX	2-hydroxy-3-keto-5-methylthiopentenyl-1-phosphate phosphatase
S19-40_00566	mtnW	2,3-diketo-5-methylthiopentyl-1-phosphate enolase
S19-40_00966	ghrB	glyoxylate/hydroxypyruvate/2-ketogluconate reductase
S19-40_01097	-	Aldo/keto reductase family protein
S19-40_01158	fadA	3-ketoacyl-CoA thiolase
S19-40_01396	-	Aldo/keto reductase family protein
S19-40_01493	csbX	Alpha-ketoglutarate permease
S19-40_01547	ilvC	Ketol-acid reductoisomerase (NADP(+))
S19-40_01623	-	Aldo/keto reductase family protein
S19-40_01653	camP	2,5-diketocamphane 1,2-monooxygenase
S19-40_01995	ydaE	putative D-lyxose ketol-isomerase
S19-40_02000	-	Aldo/keto reductase family protein
S19-40_02005	pcaR_pcaU	beta-ketoadipate pathway transcriptional regulators, PcaR/PcaU/PobR family
S19-40_02073	ulaD	3-keto-L-gulonate-6-phosphate decarboxylase UlaD
S19-40_02145	-	Aldo/keto reductase family protein
S19-40_02175	xylA	alpha-ketoglutaric semialdehyde dehydrogenase
S19-40_02310	dlgD	2,3-diketo-L-gulonate reductase
S19-40_02418	fabF	fabF: beta-ketoacyl-acyl-carrier-protein synthase II
S19-40_02521	fadA	3-ketoacyl-CoA thiolase
S19-40_02597	-	Aldo/keto reductase family protein
S19-40_02838	-	Aldo/keto reductase family protein
S19-40_02882	-	Transketolase, pyrimidine binding domain
S19-40_02895	fadA	3-ketoacyl-CoA thiolase
S19-40_03297	dxs	Transketolase, pyrimidine binding domain
S19-40_03393	scoA	putative succinyl-CoA:3-ketoacid coenzyme A transferase subunit A
S19-40_03394	scoB	putative succinyl-CoA:3-ketoacid coenzyme A transferase subunit B
S19-40_03621	scoB	putative succinyl-CoA:3-ketoacid coenzyme A transferase subunit B
S19-40_03674	kdgT	2-keto-3-deoxygluconate permease
S19-40_03678	kduI	4-deoxy-L-threo-5-hexosulose-uronate ketol-isomerase
S19-40_03822	-	Aldo/keto reductase family protein
S19-40_03832	iolI	2-keto-myo-inositol isomerase

Dehydratase related genes		
S19-40_00109	leuC	3-isopropylmalate dehydratase large subunit
S19-40_00332	sdhA	L-serine dehydratase, alpha chain
S19-40_00333	sdhB	L-serine dehydratase, beta chain
S19-40_00564	mtnB	Methylthioribulose-1-phosphate dehydratase
S19-40_00785	fabZ	3-hydroxyacyl-[acyl-carrier-protein] dehydratase FabZ
S19-40_00942	hisB	Imidazoleglycerol-phosphate dehydratase
S19-40_01000	pglF	UDP-N-acetyl-alpha-D-glucosamine C6 dehydratase
S19-40_01470	tcdA	tRNA threonylcarbamoyladenosine dehydratase
S19-40_01507	-	Prephenate dehydratase
S19-40_01531	hemB	Delta-aminolevulinic acid dehydratase
S19-40_01543	leuD1	3-isopropylmalate dehydratase small subunit 1
S19-40_01544	leuC	3-isopropylmalate dehydratase large subunit
S19-40_01736	-	NAD dependent epimerase/dehydratase family protein
S19-40_01805	-	NAD dependent epimerase/dehydratase
S19-40_02013	fabZ	3-hydroxyacyl-[acyl-carrier-protein] dehydratase FabZ
S19-40_02171	garD	Galactarate dehydratase (L-threo-forming)
S19-40_02173	gudD	Glucarate dehydratase
S19-40_02176	-	putative 5-dehydro-4-deoxyglucarate dehydratase
S19-40_02303	uxaA	Altronate dehydratase
S19-40_02308	uxuA	Mannonate dehydratase
S19-40_02782	aroD	3-dehydroquinate dehydratase
S19-40_02853	dsdA	D-serine dehydratase
S19-40_02891	prpD	2-methylcitrate dehydratase
S19-40_02926	yqhS	3-dehydroquinate dehydratase
S19-40_03226	rfbG	CDP-glucose 4,6-dehydratase
S19-40_03334	-	NAD dependent epimerase/dehydratase family protein
S19-40_03405	-	GDP-mannose 4,6 dehydratase
S19-40_03419	nnrD	ADP-dependent (S)-NAD(P)H-hydrate dehydratase
S19-40_03512	rfbB	dTDP-glucose 4,6-dehydratase
S19-40_03631	pseB	UDP-N-acetylglucosamine 4,6-dehydratase (inverting)
S19-40_03641	ilvA	L-threonine dehydratase biosynthetic IlvA
S19-40_03651	ilvD	Dihydroxy-acid dehydratase
S19-40_03828	iolE	Inosose dehydratase
S19-40_03831	iolE	Inosose dehydratase
Enoyl reductase related genes		
S19-40_00232	-	Enoyl-(Acyl carrier protein) reductase
S19-40_00327	-	Enoyl-(Acyl carrier protein) reductase
S19-40_00514	fadH	putative 2,4-dienoyl-CoA reductase
S19-40_00548	-	Enoyl-(Acyl carrier protein) reductase
S19-40_01324	-	Enoyl-(Acyl carrier protein) reductase
S19-40_01390	-	putative enoyl-[acyl-carrier-protein] reductase II
S19-40_01662	-	Enoyl-(Acyl carrier protein) reductase
S19-40_01996	-	Enoyl-(Acyl carrier protein) reductase
S19-40_02024	-	Enoyl-(Acyl carrier protein) reductase
S19-40_02139	-	Enoyl-(Acyl carrier protein) reductase
S19-40_02346	-	Enoyl-(Acyl carrier protein) reductase
S19-40_02378	fabI	Enoyl-[acyl-carrier-protein] reductase [NADH] FabI
S19-40_02513	-	Enoyl-(Acyl carrier protein) reductase
S19-40_02516	-	Enoyl-(Acyl carrier protein) reductase
S19-40_02605	-	Enoyl-(Acyl carrier protein) reductase
S19-40_02899	fadH	2,4-dienoyl-CoA reductase (NADPH2)
S19-40_03349	fabL	Enoyl-[acyl-carrier-protein] reductase [NADPH] FabL
S19-40_03372	-	Enoyl-(Acyl carrier protein) reductase
S19-40_03395	-	Enoyl-(Acyl carrier protein) reductase
S19-40_03523	-	Enoyl-(Acyl carrier protein) reductase
S19-40_03527	-	Enoyl-(Acyl carrier protein) reductase
S19-40_03679	-	Enoyl-(Acyl carrier protein) reductase
S19-40_03816	-	Enoyl-(Acyl carrier protein) reductase
Other acetyltransferase related genes		
S19-40_00053	ydaF	Putative ribosomal N-acetyltransferase YdaF
S19-40_00166	speG	Spermidine N(1)-acetyltransferase
S19-40_00220	kbl, GCAT	glycine C-acetyltransferase
S19-40_00408	ylbP	putative N-acetyltransferase YlbP
S19-40_00459	pdhC	Dihydrolipoyllysine-residue acetyltransferase component of pyruvate dehydrogenase complex
S19-40_00502	dapH	2,3,4,5-tetrahydropyridine-2,6-dicarboxylate N-acetyltransferase
S19-40_00527	-	Acetyltransferase (GNAT) family protein

S19-40_00621	-	Acetyltransferase (GNAT) domain protein
S19-40_00725	argA	amino-acid N-acetyltransferase
S19-40_00765	ywnH	Putative phosphinothricin acetyltransferase YwnH
S19-40_00936	dapH	2,3,4,5-tetrahydropyridine-2,6-dicarboxylate N-acetyltransferase
S19-40_00960	nat	Arylamine N-acetyltransferase
S19-40_01010	epsM	Putative acetyltransferase EpsM
S19-40_01045	yvbK	putative N-acetyltransferase YvbK
S19-40_01228	paiA	Spermidine/spermine N(1)-acetyltransferase
S19-40_01347	-	Acetyltransferase (GNAT) family protein
S19-40_01371	bltD	Spermine/spermidine acetyltransferase
S19-40_01402	mdpB3	acetyltransferase/esterase
S19-40_01427	oatA	O-acetyltransferase OatA
S19-40_01551	ysnE	putative N-acetyltransferase YsnE
S19-40_01659	ytmI	putative N-acetyltransferase YtmI
S19-40_01771	cysE	Serine acetyltransferase
S19-40_01853	ypeA	Acetyltransferase YpeA
S19-40_01870	yycN	putative N-acetyltransferase YycN
S19-40_01898	-	GNAT acetyltransferase
S19-40_01930	ydaF	Putative ribosomal N-acetyltransferase YdaF
S19-40_01994	ydaF	Putative ribosomal N-acetyltransferase YdaF
S19-40_02207	slyA	rimI: ribosomal-protein-alanine acetyltransferase
S19-40_02340	-	Acetyltransferase (GNAT) domain protein
S19-40_02357	ydaF	Putative ribosomal N-acetyltransferase YdaF
S19-40_02365	yjcF	putative N-acetyltransferase YjcF
S19-40_02403	yjbC	Putative acetyltransferase YjbC
S19-40_02431	argA	Amino-acid acetyltransferase
S19-40_02432	argJ	ArgJ: glutamate N-acetyltransferase/amino-acid acetyltransferase
S19-40_02456	-	Acetyltransferase (GNAT) domain protein
S19-40_02457	-	Acetyltransferase (GNAT) domain protein
S19-40_02521	yhfS	Putative acetyl-CoA C-acetyltransferase YhfS
S19-40_02525	wecD	dTDP-fucosamine acetyltransferase
S19-40_02601	phnO	Aminoalkylphosphonate N-acetyltransferase
S19-40_02798	-	rimI: ribosomal-protein-alanine acetyltransferase
S19-40_02843	mshD	Mycothiol acetyltransferase
S19-40_02845	-	ectoine_EctA: diaminobutyrate acetyltransferase
S19-40_02881	pdhC	Dihydrolipoyllysine-residue acetyltransferase component of pyruvate dehydrogenase complex
S19-40_02887	pta	Phosphate acetyltransferase
S19-40_02895	mmgA	Acetyl-CoA acetyltransferase
S19-40_03092	rimI	Ribosomal-protein-alanine acetyltransferase
S19-40_03191	-	Acetyltransferase (GNAT) family protein
S19-40_03241	-	Acetyltransferase (GNAT) family protein
S19-40_03298	pdhC	Dihydrolipoyllysine-residue acetyltransferase component of pyruvate dehydrogenase complex
S19-40_03441	patA	Peptidoglycan O-acetyltransferase
S19-40_03507	wecD	dTDP-fucosamine acetyltransferase
S19-40_03529	pta	Phosphate acetyltransferase
S19-40_03548	rimL	rimI: ribosomal-protein-alanine acetyltransferase
S19-40_03619	yodP	N-acetyltransferase YodP
S19-40_03625	rimL	rimI: ribosomal-protein-alanine acetyltransferase
S19-40_03695	yycN	putative N-acetyltransferase YycN
S19-40_03740	yjcF	putative N-acetyltransferase YjcF
S19-40_03744	-	Acetyltransferase
S19-40_03746	wecD	dTDP-fucosamine acetyltransferase
S19-40_03758	maa	Maltose O-acetyltransferase
S19-40_03848	ytmI	putative N-acetyltransferase YtmI
S19-40_03930	cysE	Serine acetyltransferase
S19-40_03976	glmU	glmU: UDP-N-acetylglucosamine diphosphorylase/glucosamine-1-phosphate N-acetyltransferase
