# Supplementary material for: Genome sequence analysis of the beneficial Bacillus subtilis PTA-271 isolated from a Vitis vinifera (cv. Chardonnay) rhizospheric soil: assets for sustainable biocontrol
Source: Environ Microbiome. 2021 Jan 29;16:3. doi: 10.1186/s40793-021-00372-3 (PMC8067347; doi:10.1186/s40793-021-00372-3)
Supplement: Supplementary file 8 — Additional file 8: Table S8. Anti-SMASH 5.1.0 prediction of gene clusters responsible for secondary metabolite production in Bacillus subtilis PTA-271. [file 40793_2021_372_MOESM8_ESM.pdf]

Table S8: Anti-SMASH 5.1.0 prediction of gene clusters responsible for secondary metabolite production in *Bacillus subtilis* PTA-271.

Secondary metabolite	Enzyme	Gene clusters	Compound	Similarity
Betalactone-NRPS	NRPS	1	Fengycin	100%
NRPS	NRPS	2	Bacillibactin	100%
			Surfactin	78%
Other	NRPS	1	Bacilysin	100%
T3PKS	PKS	1	-	0%
TransATPKS-NRPS-PKSlike	PKS/NRPS	1	Bacillaene	100%
tRNA-dependent cyclodipeptide synthases	-	1	-	0%
Terpene	-	2	-	0%
Sactipeptide	RIPP	2	Subtilosin A	100%
			Sporulation killing factor	100%
